# Suppression of KSHV lytic replication and primary effusion lymphoma by selective RNF5 inhibition

**DOI:** 10.1371/journal.ppat.1011103

**Published:** 2023-01-19

**Authors:** Xiaojuan Li, Fan Wang, Xiaolin Zhang, Qinqin Sun, Ersheng Kuang

**Affiliations:** 1 Institute of Human Virology, Zhongshan School of Medicine, Sun Yat-Sen University, Guangzhou, China; 2 College of Clinical Medicine, Hubei University of Chinese Medicine, Wuhan, China; 3 Key Laboratory of Tropical Disease Control (Sun Yat-Sen University), Ministry of Education, Guangzhou, China; Vanderbilt University Medical Center, UNITED STATES

## Abstract

Primary effusion lymphoma (PEL), a rare aggressive B-cell lymphoma in immunosuppressed patients, is etiologically associated with oncogenic γ-herpesvirus infection. Chemotherapy is commonly used to treat PEL but usually results in poor prognosis and survival; thus, novel therapies and drug development are urgently needed for PEL treatment. Here, we demonstrated that inhibition of Ring finger protein 5 (RNF5), an ER-localized E3 ligase, suppresses multiple cellular pathways and lytic replication of Kaposi sarcoma-associated herpesvirus (KSHV) in PEL cells. RNF5 interacts with and induces Ephrin receptors A3 (EphA3) and EphA4 ubiquitination and degradation. RNF5 inhibition increases the levels of EphA3 and EphA4, thereby reducing ERK and Akt activation and KSHV lytic replication. RNF5 inhibition decreased PEL xenograft tumor growth and downregulated viral gene expression, cell cycle gene expression, and hedgehog signaling in xenograft tumors. Our study suggests that RNF5 plays the critical roles in KSHV lytic infection and tumorigenesis of primary effusion lymphoma.

## Author summary

Primary effusion lymphoma is a rare malignant B-cell lymphoma with multifarious gene mutations and genomic abnormalities, and is tightly associated with KSHV infection; currently, no effective therapy for PEL has been established. RNF5 regulates autophagy, antiviral and antitumor immunity, inflammation and metabolism, which are important for tumorigenesis. However, whether RNF5 acts as a therapeutic target for viral infection or cancer chemotherapy remains unclear. Here, we demonstrated that RNF5 inhibition suppresses PEL xenograft tumor growth and oncogenic KSHV lytic replication through increased EphA3 and EphA4 levels and decreased downstream pathways. Thus, our study may provide a promising candidate for developing the treatment of KSHV-positive diseases and primary effusion lymphoma.

## Introduction

Primary effusion lymphoma (PEL) is a rare aggressive B-cell lymphoma in immunosuppressed patients, such as AIDS patients and post-transplant patients. PEL is etiologically associated with infection with Kaposi sarcoma-associated herpesvirus (KSHV) and mostly occurs with co-infection with Epstein-Barr virus [1–3]. Currently, no appropriate and effective therapy has been established for PEL. Chemotherapy is commonly used to treat PEL patients, but resistance rapidly develops; thus, traditional chemotherapy usually results in poor prognosis and survival. Many gene mutations have been identified in PEL, including a conserved mutation of interleukin 1 receptor-associated kinase 1 (IRAK1) found in all PELs which results in constitutively active myeloid differentiation factor 88 (MyD88)/IRAK1 signaling for the development and progression of PEL [4, 5]. Many preclinical targeting therapeutic strategies have been developed for PEL treatment, including inhibition of cellular signaling and elimination of viral infections [3, 6–8], providing a promising choice for novel PEL therapies and drug development that are urgently needed for PEL treatment.

Ephrin receptors are a family of receptor tyrosine kinases (RTKs) of transmembrane ephrins that act as ligands [9, 10]. Fourteen members are divided into two subtypes according to their ligands: nine type-A members recognize four Ephrin-A ligands, and five type-B members bind to three Ephrin-B ligands. They play bidirectional roles in many important physiological processes in a ligand-independent or ligand-dependent manner, including growth, development, differentiation and tumorigenesis [11]. Ephrin receptors and ligands are widely expressed in different cells and tissues. Their expression is often discordantly changed in tumors and stromal cells, triggering an imbalance of receptors-ligands and dysfunction of downstream signaling in cancer progression.

During KSHV infection, three types of Ephrin type-A receptors (EphA2, EphA4, and EphA7) have been identified as receptors for KSHV entry and fusion in endothelial cells and B cells [12–15]. Ephrin receptors and ligands also play important roles in KSHV pathogenesis; EphA2 and EphA4 receptors and Ephrin-B2 ligands are abundantly expressed in KS cell lines and tumor biopsies, and Ephrin-B2 acts as an essential factor in KS cell viability and tumor growth [16, 17]. However, their regulation, function, and mechanism in KSHV lytic replication and pathogenesis remain largely unknown.

Ring finger protein 5 (RNF5), also known as RMA1, is an 18 kDa E3 ligase localized on the cytoplasmic surface of the endoplasmic reticulum and is ubiquitously expressed in diverse cells and tissues, with the highest expression in breast cancer and melanoma [18]. This protein interacts with several substrates and induces their ubiquitination and degradation to regulate multiple physiological processes, including autophagy, antiviral and anti-tumor immunity, inflammation, and metabolism [19–24], which are important for tumorigenesis and drug responsiveness in cancer chemotherapy. Although increased RNF5 expression might be commonly associated with tumor progression and decreased survival [18, 25–27], the opposite findings have been reported in malignant breast cancers [21, 28]; however, the latter are related to a p53-mutated background. Recently, the tumor-promoting functions of RNF5 in the development and survival of acute myeloid leukemia (AML) have been revealed. Inhibition of RNF5 can suppress AML cell growth and improve the responsiveness to HDAC inhibitors [29], indicating that RNF5 could act as a candidate target for chemotherapy of leukemia and lymphoma. A specific and selective RNF5 inhibitor, INH2, has been developed and provides a promising approach for further investigation of RNF5 function and therapeutic potential in cancers and other diseases [30].

KSHV primary infection and lytic replication induce robust ERK and Akt activation through multiple viral products and different mechanisms, and inhibition of both pathways can suppress KSHV lytic replication and reactivation from latency [31–36], indicating that both pathways are required for KSHV lytic replication and pathogenesis. Since PEL constitutively activates the phosphatidylinositide 3-kinase (PI3K)-Akt and extracellular regulated kinase (ERK)-MAPK pathways, we investigated the role of RNF5 in lytic replication and the tumor-suppressive effect of RNF5 inhibition in PEL using different approaches. We found that selective RNF5 inhibition suppresses KSHV lytic replication and PEL tumorigenesis by increasing EphA3 and EphA4 levels and then disrupting ERK and Akt activation, hedgehog and cell cycle gene expression, and KSHV lytic replication, indicating that RNF5 plays the essential roles in KSHV lytic infection and PEL treatment.

## Results

### RNF5 inhibition decreases multiple cellular pathways and KSHV lytic replication in PEL cells

**Fig 1:**
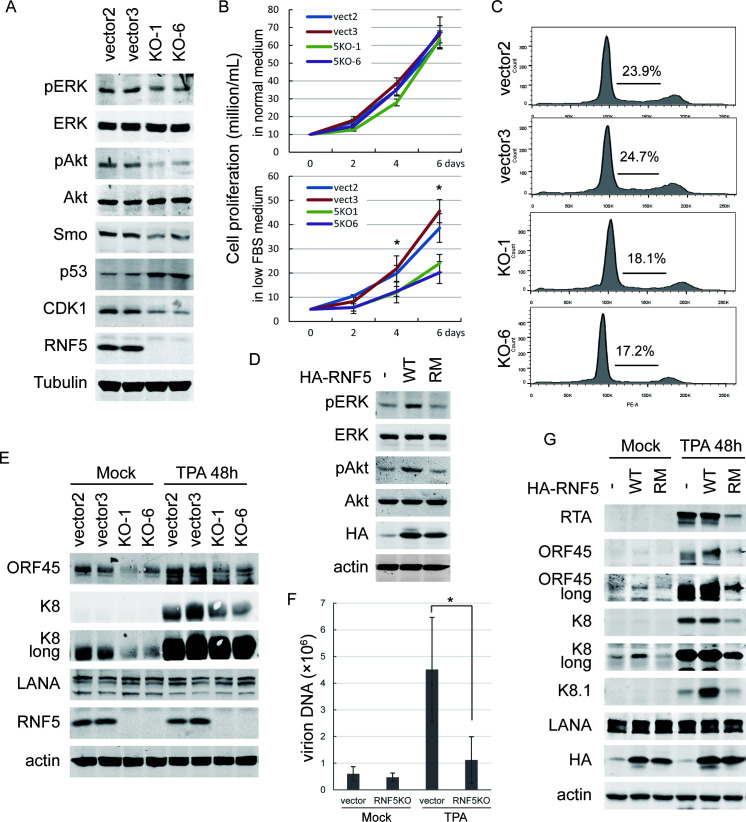
RNF5 loss decreases ERK and Akt activation, CDK1 and Smo expression and KSHV lytic replication in PEL cells. A. CRISPR/Cas9-mediated control or RNF5 KO BCBL1 cells were collected, and cell extracts were analyzed by western blotting for ERK and Akt phosphorylation levels and Smo, CDK1 and p53 expression levels. B. The proliferation of control or RNF5 KO BCBL1 cells was measured in normal medium containing 10% FBS (top) or in low serum medium containing 2% FBS (bottom). After counting the living cells, the means of cell numbers from three independent experiments were recorded, and the growth curves are shown. *, p<0.05. C. The cell cycle of control vs. RNF5 KO BCBL1 cells was measured by flow cytometry after PI staining, the representative images of cell cycle and the percentage of S phase of two independent experiments are shown. D. Stable BCBL1 cells transduced with empty vector, RNF5 WT or E3 ligase-inactivated RING domain-mutated (RM) construct were collected, and cell lysates were detected by western blotting for ERK and Akt phosphorylation. E. Control or RNF5 KO BCBL1 cells were left untreated or induced by TPA for 48 h, and then cells were collected, and cell extracts were analyzed by western blotting for viral gene expression levels. F. The virions in supernatants of control or RNF5 KO BCBL1 cells were collected after TPA induction for 4 days, and then virion DNA was isolated and detected by real-time PCR. *, p<0.05. G. BCBL1 cells were stably transduced with empty vector, RNF5 WT or RM construct, and then were left untreated or induced by TPA for 48 h. The cell lysates were subjected to western blotting analysis for viral gene expression levels.

To determine the effect of RNF5 inhibition in PEL cells, we established two independent cell lines with CRISPR-Cas9-mediated RNF5 knockout (KO) in BCBL1 cells and two control cells with empty vector transduction (Fig 1A). These cells did not exhibit an obvious difference in cell morphology and had normal proliferation compared with the control cells under normal culture condition (Fig 1B, top), whereas RNF5 KO BCBL1 cells showed decreased proliferation under low serum conditions compared with the control cells (Fig 1B, bottom), indicating that RNF5 KO decreased the independence of serum or growth factors in PEL cell growth. The cell cycle was further analyzed in these cells by propidium iodide (PI) staining and flow cytometry, and the percentage of cells in the S phase was lower in RNF5 KO cells than in RNF5 control cells (Fig 1C). The results suggest that RNF5 loss reduces cell growth under low-serum conditions.

To investigate whether the cellular signaling pathways were affected by RNF5 loss, activation of the ERK-MAPK and PI3K pathways was detected in BCBL1 control vs. RNF5 KO cells. Decreased ERK and Akt phosphorylation was observed in RNF5 KO cells compared to control cells expressing RNF5 (Fig 1A). In contrast, ectopic overexpression of the RNF5 wild-type (WT) construct increased ERK and Akt phosphorylation in BCBL1 cells, whereas the E3 ligase-inactivated RNF5 RING domain-mutated (RM) construct exhibited minimal effects (Fig 1D). These results suggest that RNF5 inhibition results in decreased ERK and Akt activation in the PEL cells.

Previous studies have shown that both ERK and Akt activation are essential for KSHV lytic replication [35, 37, 38]. We further investigated whether KSHV gene expression was affected by RNF5 loss in BCBL1 cells. Approximately 5% of BCBL1 cells undergo ORF45-positive spontaneous lytic replication [39], low spontaneous expression of KSHV lytic genes was observed in BCBL1 cells, and the 12-O-tetradecanoylphorbol-13-acetate (TPA) treatment greatly induced lytic gene expression [40]. Both spontaneous and TPA-induced lytic gene expression levels of ORF45 and K8 were decreased in RNF5 KO BCBL1 cells compared with control cells (Fig 1E), whereas the latent gene expression level of LANA was not affected. Consistently, virion production was greatly decreased in RNF5 KO cells compared with control cells (Fig 1F). Furthermore, ectopic RNF5 WT overexpression increased the lytic gene expression levels of the immediate early gene ORF45 and late gene K8.1 in BCBL1 cells under TPA induction, whereas the RNF5 RM construct did not (Fig 1G), and no change in LANA expression level was observed under either condition (Fig 1E and 1G). Notably, the spontaneous expression of ORF45 and K8 was also increased by RNF5 WT overexpression, but not by RNF5 RM overexpression. These results suggest that RNF5 inhibition suppresses both spontaneous and TPA-induced KSHV lytic gene expression and replication.

Studies have revealed that RNF5 silencing increases tumor suppressor p53 levels in breast cancer cells [18], and RNF5 KO cells exhibited a decreased cell cycle progression (Fig 1C); thus, we investigated whether the expression of p53 and cell cycle genes were affected in RNF5 KO cells. As expected, p53 levels were increased, while cyclin-dependent kinase 1 (CDK1) was decreased in RNF5 KO BCBL1 cells compared with control cells (Fig 1A). Interestingly, ERK-MAPK signaling can positively regulate hedgehog gene expression [41, 42], and the level of Hedgehog receptor transducer Smoothened (Smo) was decreased following decreased ERK activation in RNF5 KO BCBL1 cells compared with control cells (Fig 1A). Similarly, in RNF5 KO MEFs, the expression levels of Smo and CDK1 were decreased (S1 Fig). These results suggest that RNF5 loss commonly decreases Smo and CDK1 expression. To further investigate the pathways required for Smo and CDK1 suppression by RNF5 loss, we treated RNF5 WT and KO MEFs with different drugs and detected Smo and CDK1 expression. We found that RNF5 KO significantly decreased Smo and CDK1 expression levels, and ER stress induced by DTT treatment partially recovered their expression levels in RNF5 KO MEFs. Treatment with the MEK inhibitor U0126 decreased the expression levels of both genes in RNF5 WT cells to a level equal to that in RNF5 KO cells, whereas autophagic inhibition by chloroquine treatment barely affected their expression (S1 Fig). These results indicate that inhibition of RNF5 suppresses Smo and CDK1 expression by decreasing ERK activation, but not through autophagic induction.

### RNF5 inhibitor suppresses cellular pathways and viral lytic gene expression in PEL cells

**Fig 2:**
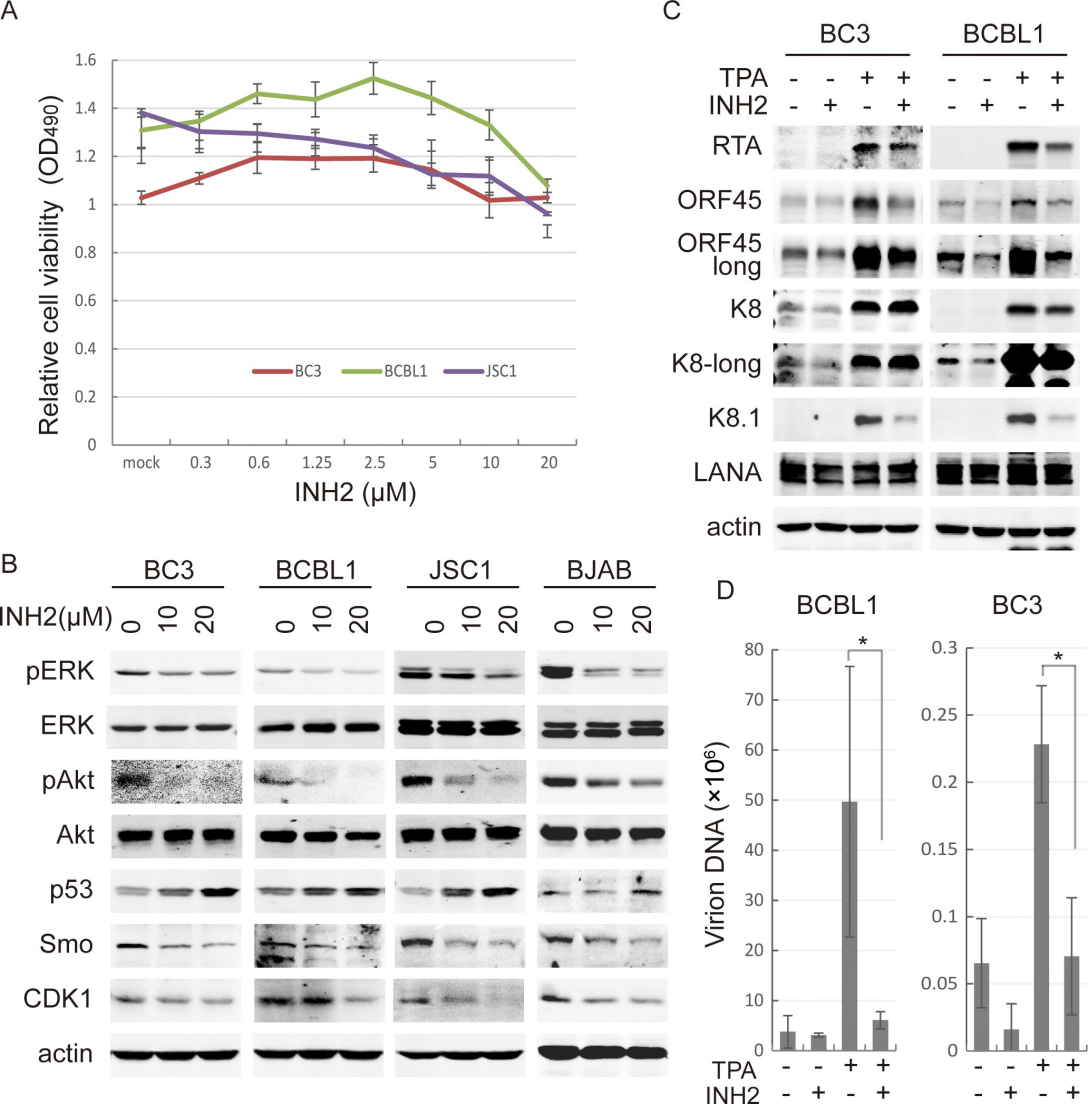
RNF5 inhibitor suppresses ERK and Akt activation, p53, CDK1 and Smo expression and KSHV lytic replication in PEL cells. A. BC3, BCBL1 and JSC1 cells were treated with different concentrations of INH2 for 72 h. The relative cell viability was determined by MTS assay. The mean ± SD was calculated from three independent experiments in triplicate, and the curves of cell viability are shown. B. BC3, BCBL1, JSC1 and BJAB cells were treated with 0, 10 or 20 μM INH2 for 72 h, and the phosphorylation levels of ERK and Akt and the expression levels of p53, CDK1 and Smo were detected by western blotting as indicated. C. BC3 and BCBL1 cells were left untreated or induced by TPA for 72 h in the absence or presence of 10 μM INH2. The cells were collected, and whole cell extracts were detected by western blotting for viral gene expression. D. The supernatant of BC3 or BCBL1 cells was collected after TPA induction for 4 days, and then virion DNA was isolated and detected by real-time PCR. *, p<0.05.

To further investigate the function of RNF5 inhibition in PEL cells, three KSHV-positive PEL cell lines, namely, BC3, BCBL1 and JSC1, were treated with the RNF5 inhibitor INH2 [30]. The viability of the three cell types was slightly affected by the RNF5 inhibitor (Fig 2A), indicating that INH2 did not exhibit obvious cell toxicity in PEL cells. Interestingly, INH2 treatment decreased ERK and Akt phosphorylation in the three types of PEL cells in a dose-dependent manner (Fig 2B). To confirm the effect of RNF5 inhibition on cellular gene expression levels, including the cell cycle and hedgehog genes, we analyzed the expression of these genes and found that INH2 decreased the levels of Smo and CDK1 and increased p53 level in a dose-dependent manner (Fig 2B). To exclude the possibility that the inhibition of cellular signaling by the RNF5 inhibitor was dependent on viral gene expression, KSHV-negative lymphoma cells BJAB were treated with INH2, and similar inhibition of ERK and Akt phosphorylation, decreased Smo and CDK1 expression and increased p53 level were observed under INH2 treatment (Fig 2B). Thus, we concluded that the RNF5 inhibitor significantly inhibited multiple cellular pathways in the PEL cells.

Furthermore, INH2 inhibited spontaneous ORF45 and K8 expression in both BC3 and BCBL1 cells, and greatly decreased TPA-induced expression of immediate early genes ORF45 and RTA and late gene K8.1 in both cells, moderately reduced K8 expression in BCBL1 cells, and slightly reduced it in BC3 cells (Fig 2C). Virion production was similarly suppressed by INH2 treatment in both cell lines after TPA induction, with approximate 85% inhibition in BCBL1 cells and 70% inhibition in BC3 cells (Fig 2D), suggesting that the RNF5 inhibitor suppressed both spontaneous and TPA-induced KSHV lytic replication in PEL cells.

### RNF5 downregulates the levels of EphA3 and EphA4 in PEL cells

**Fig 3:**
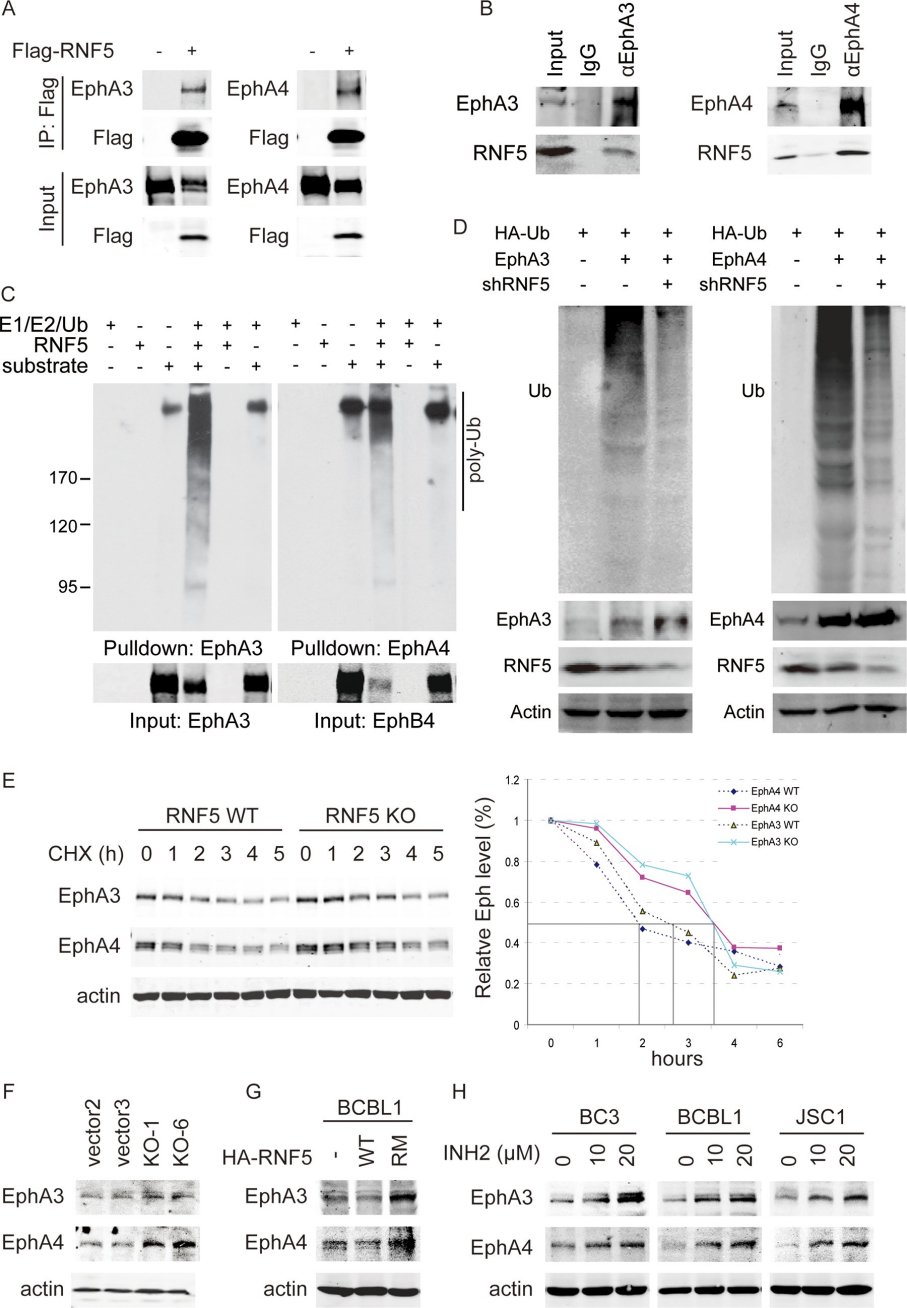
RNF5 interacts with EphA3 and EphA4 and induces their ubiquitination and degradation. A. The empty vector or Flag-tagged RNF5-expressing plasmid was cotransfected into 293T cells with EphA3- or EphA4-expressing plasmids for 48 h. The cells were collected, whole cell extracts were subjected to immunoprecipitation (IP) with anti-Flag antibody, and then IP complexes were detected by western blotting as indicated. B. Whole cell extracts from iSLK.219 cells or BCBL1 cells were subjected to IP with anti-EphA3 or anti-EphA4 antibody, respectively, with normal mouse IgG as control, and then IP complexes were detected by western blotting as indicated. C. EphA3 or EphA4 proteins were affinity-purified from 293T cells transfected with EphA3- or EphA4-expressing plasmid for 48 h and then added to an in vitro ubiquitination reaction as a substrate with purified RNF5 as the E3 ligase. After stopping the reaction by adding 1% SDS, the complexes were analyzed by western blotting with anti-Ub antibody. D. HEK293 cells were transfected with EphA3 or EphA4-expressing plasmid and transduced with shRNF5 or scramble shRNA for 48h, and then the cells were collected and lysed in presence of final 1% SDS. After 10 fold dilution to final 0.1% SDS, EphA3 or EphA4 protein were immunoprecipitated with anti-EphA3 or anti-EphA4 antibody, respectively, and in vivo ubiquitination of EphA3 or EphA4 were detected with anti-Ub antibody. E. RNF5 wild-type or KO MEFs were pulse treated with cycloheximide (CHX) for different times, and then cells were collected, lysed, and analyzed by western blotting as indicated. The mean density of EphA3 or EphA4 bands was quantitated in three independent western blots using ImageJ software, and the half-life curves of EphA3 or EphA4 are shown. F-G. Stable control or RNF5 KO BCBL1 cells (F) and stable BCBL1 cells transduced with empty vector, RNF5 WT or RM constructs (G) were collected, and the cell extracts were detected by western blotting for EphA3 and EphA4 levels. H. Three kinds of PEL cells were treated with 0, 10 or 20 μM INH2 or solvent for 72 h, and the expression levels of EphA3 and EphA4 were detected in these cell extracts by western blotting.

Three types of Ephrin-type A receptors (EphA2, EphA4 and EphA7) serve as KSHV receptors [12, 13, 15], and our preliminary mass spectrometry analysis revealed that RNF5 associates with Ephrin-type A receptors. To investigate the mechanism of RNF5 regulation in PEL cells, we detected the interaction between RNF5 and ephrin type-A receptors using immunoprecipitation and found that ectopic RNF5 interacted with overexpressed EphA3 and EphA4 in 293T cells (Fig 3A). Endogenous RNF5-EphA3 and RNF5-EphA4 interactions were further observed in iSLK.219 and BCBL1 cells, respectively (Fig 3B). As a ubiquitin E3 ligase, RNF5 directly induced EphA3 and EphA4 ubiquitination in vitro (Fig 3C), and RNF5 depletion in 293T cells by shRNF5 greatly decreased their ubiquitination (Fig 3D), suggesting that EphA3 and EphA4 act as two substrates of RNF5 for ubiquitination. Subsequently, the half-lives of EphA3 and EphA4 were prolonged in RNF5 KO MEFs compared with RNF5 WT MEFs (approximately from 2.5 and 2 to 3.5 h, respectively) (Fig 3E). These results suggested that RNF5 interacts with EphA3 and EphA4 and induces their ubiquitination and degradation.

The effects of RNF5 on ephrin type-A receptors were detected in RNF5 WT BCBL1 cells compared to RNF5 KO BCBL1 cells. Real-time PCR analysis showed that EphA4 was highly expressed and EphA3 was moderately expressed in BCBL1 cells without EphA2 expression (S2 Fig), and increased EphA3 and EphA4 protein levels were observed in RNF5 KO BCBL1 cells compared with the control cells (Fig 3F). RNF5 WT overexpression decreased their levels, while the catalytic activity-dead RNF5 RM construct increased them (Fig 3G). Alternatively, the RNF5 inhibitor INH2 increased the levels of EphA3 and EphA4 in three types of PEL cell lines (BC3, BCBL1 and JSC1). BCBL1 and JSC1 cells exhibited a strong increase in the presence of INH2 treatment, and BC3 cells showed a moderate increase under INH2 treatment (Fig 1H). These results suggested that RNF5 inhibition could upregulate the levels of EphA3 and EphA4 in PEL cells.

### EphA3 and EphA4 are essential for the downregulation of ERK and Akt activation and lytic viral gene expression in RNF5 KO PEL cells

**Fig 4:**
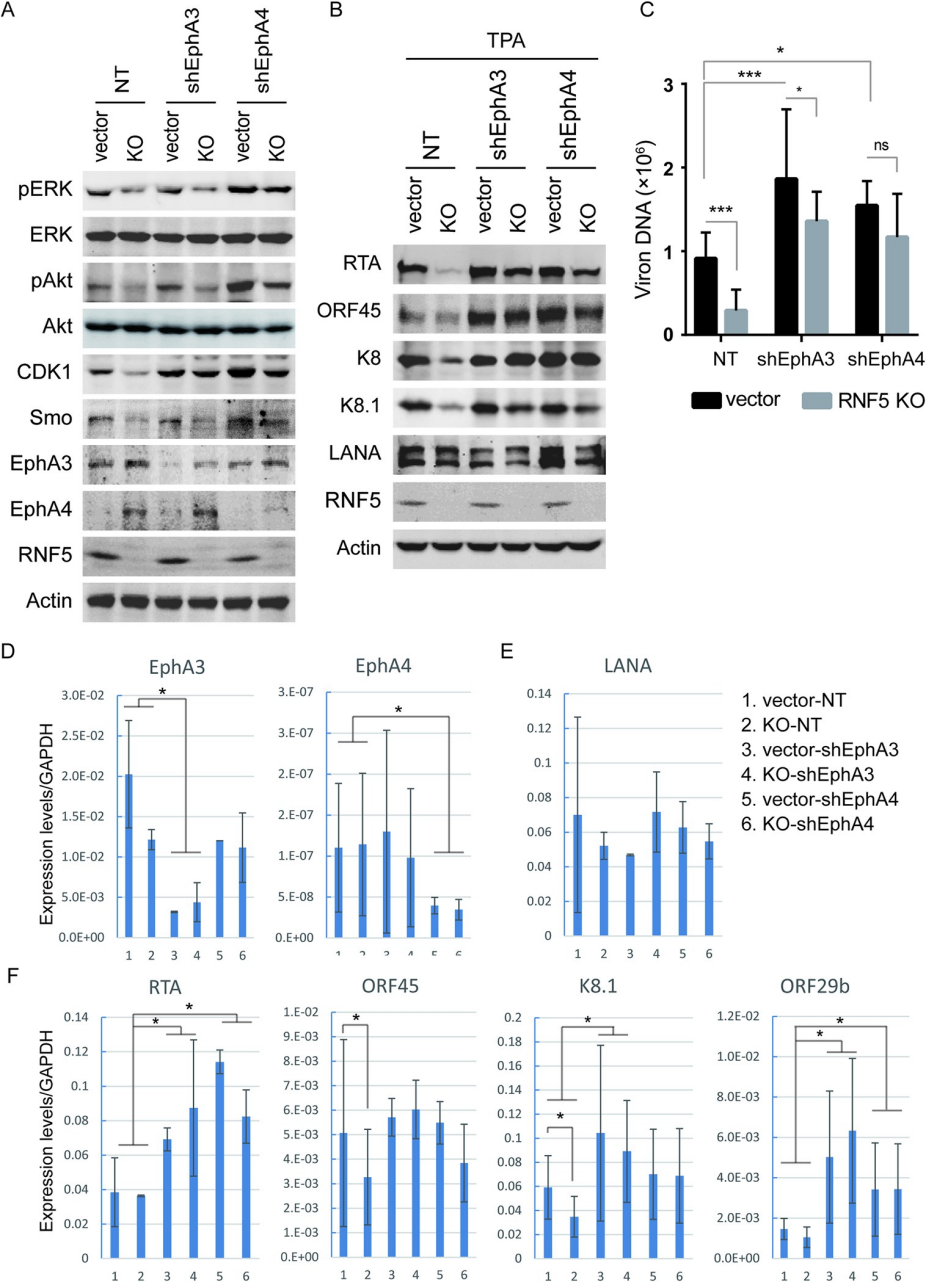
Depletion of EphA3 or EphA4 recovers the decreased ERK and Akt activation and lytic gene expression in RNF5 KO PEL cells. Control or RNF5 KO BCBL1 cells were infected with lentiviruses expressing non-target (NT) shRNA, EphA3 shRNA, or EphA4 shRNA for 48 h. A. The cells were collected, and cell lysates were analyzed by western blotting for Akt and ERK phosphorylation and CDK and Smo expression levels. B. The cells were left untreated or treated with TPA for 72 h, collected, and cell extracts were subjected to western blotting for viral gene expression. C. Cells were treated with TPA for 4 days, supernatants were collected, and extracellular virion production was detected by real-time PCR. *, p<0.05; ***, p<0.001; ns, no statistically significant differences were observed. D. Total RNA was extracted and reverse-transcribed to cDNA, and the RNA levels of EphA3 and EphA4 were detected using real-time PCR and normalized to GAPDH. *, p<0.05. E-F. The expression levels of the latent gene LANA (E) and the immediate early viral genes and late viral genes (F) were detected by real-time PCR and normalized to GAPDH. The mean ± SD was calculated from two independent cell lines in each group in triplicate. *, p<0.05.

To further measure the roles of EphA3 and EphA4 in RNF5 inhibition in PEL cells, their expression levels were depleted using shRNA in control and RNF5 KO BCBL1 cells (Fig 4A). Since BCBL1 exhibited high EphA4 expression and low EphA3 expression (S2 Fig), the decreased ERK and Akt phosphorylation was mainly recovered by EphA4 shRNA and minimally recovered by EphA3 shRNA in RNF5 KO cells compared to control cells (Figs 4A and S3A). These results suggest that RNF5 mainly downregulates EphA4 and in turn upregulates ERK and Akt activation in BCBL1 cells. Next, we investigated downstream cellular gene expression after EphA3 or EphA4 knockdown in RNF5 WT and KO BCBL1 cells. The decreased CDK1 and Smo expression in RNF5 KO cells was greatly recovered by EphA4 depletion, and they were moderately recovered by EphA3 depletion (Figs 4A and S3A), indicating that the decreased CDK1 and Smo expression levels were similarly due to an increase in EphA3 and EphA4 levels in RNF5 KO BCBL1 cells.

When KSHV lytic and latent gene expression was investigated in RNF5 WT vs. KO BCBL1 cells under TPA induction, the decreased levels of immediate early genes (RTA, ORF45, and K8) and late gene K8.1 in RNF5 KO cells were recovered under either EphA3 depletion or EphA4 depletion, whereas the level of latent gene LANA was not affected (Figs 4B and S3A). Similarly, depletion of EphA3 or EphA4 increased virion production (with 2.04 or 1.69 fold, respectively), and the decreased virion production in RNF5 KO cells was restored by EphA3 shRNA alone or EphA4 shRNA alone, approximate 70% inhibition in RNF5 KO cells was attenuated to less than 30% by EphA3 or EphA4 depletion (Fig 4C). However, neither decreased ERK/Akt phosphorylation nor lytic gene expression was recovered by EphrinA1 or EphrinA4 treatment for 3 days in RNF5 KO BCBL1 cells compared with control BCBL1 cells (S4 Fig), indicating that RNF5 regulates ERK/Akt phosphorylation and KSHV lytic replication through ligand-independent EphA3/EphA4 function. Next, we investigated whether spontaneous lytic gene expression in RNF5 WT vs. KO cells was affected by EphA3 or EphA4 depletion. Although latent gene LANA expression was not affected by either RNF5 loss or EphA3 and EphA4 depletion (Fig 4D and 4E), the decreased lytic gene expression levels of ORF45 and K8.1 were recovered by EphA3 depletion or EphA4 depletion in RNF5 KO BCBL1 cells compared with control cells, and the lytic genes RTA and ORF29b were activated by either EphA3 depletion or EphA4 depletion (Fig 4F). These results suggested that RNF5 loss downregulates spontaneous KSHV lytic gene expression by increasing EphA3 and EphA4 levels. Therefore, RNF5 inhibition increased the levels of EphA3 and EphA4, consequently decreasing ERK and Akt activation and downregulating viral lytic gene expression in PEL cells.

### RNF5 inhibition decreases ERK and Akt activation and KSHV lytic replication through increased EphA3 and EphA4 levels

**Fig 5:**
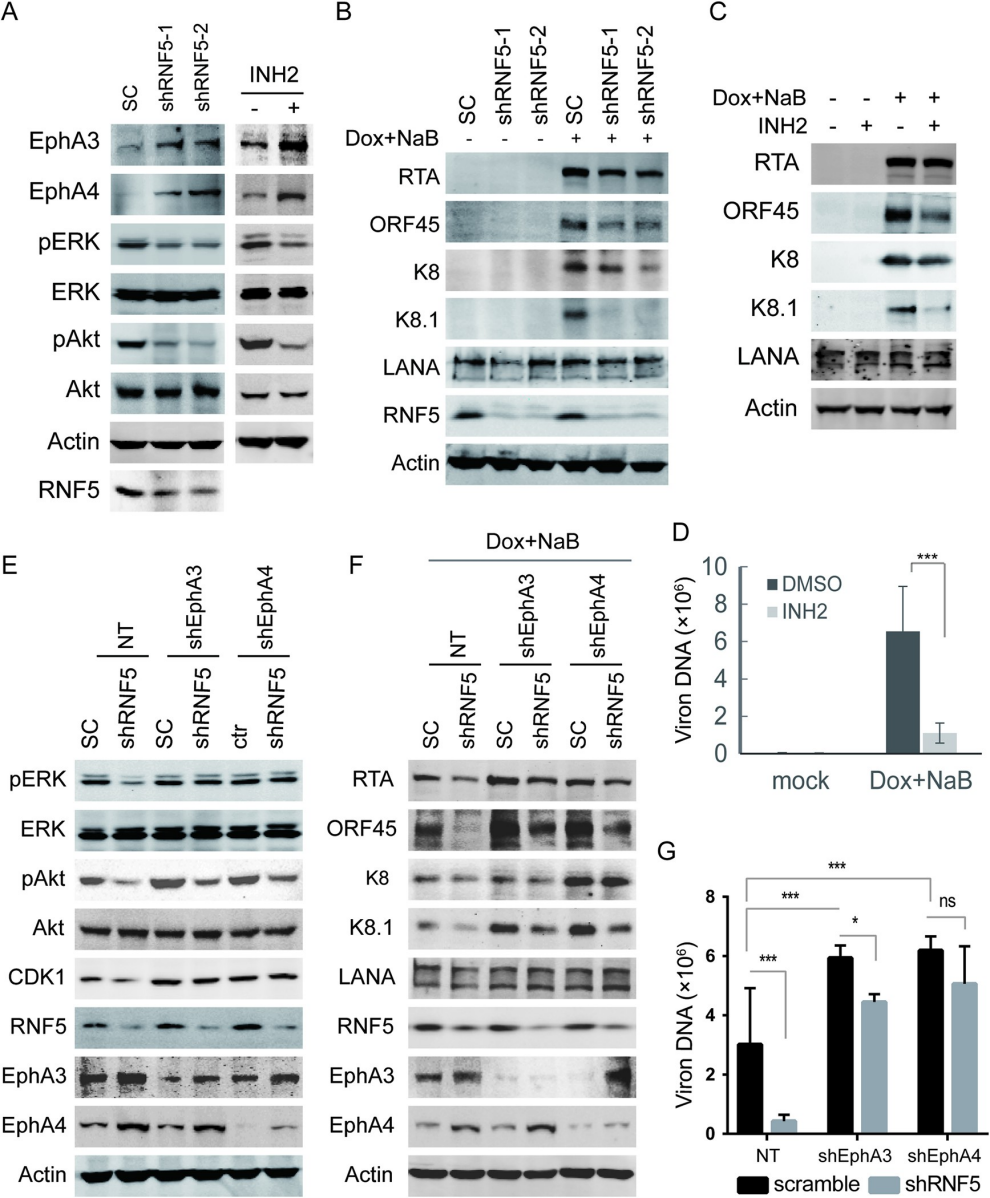
RNF5 depletion decreases cellular gene expression and lytic replication through increased EphA3 and EphA4 level in iSLK.219 cells. A-B. iSLK.219 cells were infected with scramble (SC) or shRNF5-expressing lentiviruses for 72 h, or iSLK.219 cells were left untreated or treated with 10 μM INH2 for 72h, and then cells were collected and cell extracts were subjected to western blotting analysis as indicated for ERK and Akt phosphorylation and cellular gene expression (A). The cells were left untreated or induced by Dox plus NaB for additional 72 h, the cell extracted were subjected to western blots to detect viral gene expression (B). C-D. iSLK.219 cells were left untreated or treated with 10 μM INH2, and left untreated or induced by Dox plus NaB for 72 h, the cell extracts were subjected to western blotting analysis as indicated for cellular and viral gene expression (C). After Dox plus NaB induction for 5 days, the extracellular virion production was detected in three independent experiments and virion production were shown as mean ± SD (D). ***, p<0.001. E-G. iSLK.219 cells were double infected with lentiviruses that expresses scramble or shRNF5, non-target or shEphA3 or shEphA4 as indicated for 48 h. The cells were collected and ERK and Akt phosphorylation and cellular gene expression were detected as described above (E). After cells were induced by Dox plus NaB for additional 72 h, viral gene expression were detected as described above (F). After cells were induced by Dox plus NaB for 5 days, the extracellular virion DNA were extracted and detected by real-time PCR in three independent experiments and virion production were shown as mean ± SD (G). *, p<0.05; **, p<0.01; ***, p<0.001; ns, no statistical difference.

Next, we investigated whether RNF5 inhibition suppresses cellular signaling and KSHV lytic replication in doxycycline (Dox)-inducible KSHV lytic infection to exclude the possibility that TPA has profound effects and that RNF5 inhibition directly disrupts TPA-related signaling. iSLK.219 cells were transduced with scramble shRNA or shRNF5, and then RNF5 depletion greatly increased EphA3 and EphA4 levels compared with scramble shRNA transduction, resulting in the decreased ERK and Akt phosphorylation (Fig 5A, left). The treatment with RNF5 inhibitor in iSLK.219 cells also increased EphA3 and EphA4 levels, decreased ERK and Akt phosphorylation (Fig 5A, right). As results, the lytic gene expression (ORF45, K8 and K8.1) induced by Dox+NaB treatment was dramatically decreased in RNF5 depleted cells compared with control cells, RTA expression was also slightly reduced whereas latent gene LANA expression was not affected (Fig 5B). Similarly, the RNF5 inhibitor suppressed KSHV lytic gene expression but not latent gene expression in iSLK.219 cells (Fig 5C), and consequently, the RNF5 inhibitor reduced virion production (Fig 5D). These results suggest that RNF5 depletion or inhibition commonly increases EphA3 and EphA4 levels, decreases ERK and Akt activation, and then suppresses KSHV lytic replication.

To further confirm the roles of EphA3 and EphA4 in RNF5 inhibitory function, the expression of EphA3 or EphA4 was depleted by shRNA transduction in iSLK.219 cells. The decreased ERK and Akt phosphorylation in RNF5 depleted cells was restored by either EphA3 silence or EphA4 silence (Figs 5E, top and S3B), indicating that RNF5 depletion suppressed ERK and Akt activation by increasing EphA3 and EphA4 levels. Similarly, the CDK1 expression was decreased by RNF5 knockdown in iSLK.219 cells with undetectable p53 expression, while EphA3 depletion or EphA4 depletion also restored CDK1 expression (Fig 5E, middle). Consequently, the lytic gene expression in RNF5 knockdown cells was also restored by EphA3 depletion or EphA4 depletion (Figs 5F and S3B), the virion production was increased approximately in 2 fold by EphA3 depletion or EphA4 depletion, and the decrease of virion production in RNF5 knockdown cells was similarly recovered by either depletion compared with the control cells, approximately 85% inhibition in RNF5 depleted cells was restored to 25% or 18% under EphA3 depletion or EphA4 depletion, respectively (Fig 5G). These results conclude that RNF5 silence commonly suppresses KSHV lytic replication and downregulates ERK and Akt activation by increasing EphA3 and EphA4 levels.

### RNF5 inhibition decreases PEL xenograft tumor growth

**Fig 6:**
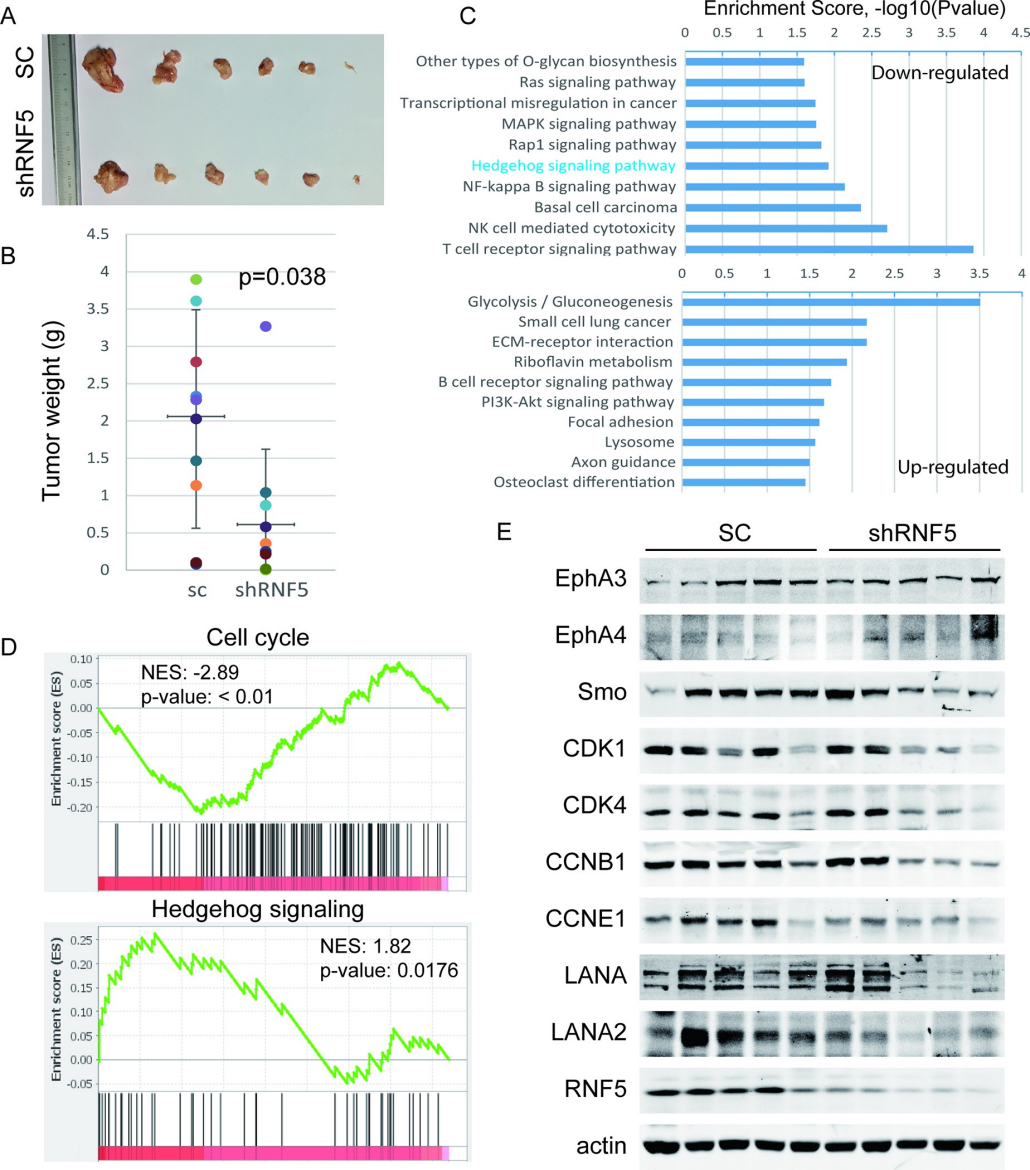
RNF5 depletion suppresses PEL xenograft tumor growth and decreases the levels of cell cycle gene expression and hedgehog signaling in tumors. A-B. Stable scramble or shRNF5-transduced BCBL1 cells were subcutaneously injected into NOD/SCID mice for 6 weeks. After the mice were sacrificed and the tumors were collected, representative images of tumors are shown (A), the tumor weights were measured, and the mean ± SD are shown (B). C. Total RNA was extracted from xenograft tumors derived from scramble or shRNF5-transduced BCBL1 cells and analyzed by Agilent Human GE 4x44k v2 microarrays. The top 10 upregulated or downregulated pathways involved in tumorigenesis are shown. D. The cell cycle and hedgehog signaling pathways were enriched in the transcriptional profile of scramble or shRNF5-transduced xenograft tumors by GSEA. E. Whole extracts from 5 random tumor samples derived from scramble or shRNF5-transduced BCBL1 cells were analyzed by western blotting as indicated for the expression of EphA3 and EphA4, Smo and cell cycle genes as well as viral latent gene expression.

**Fig 7:**
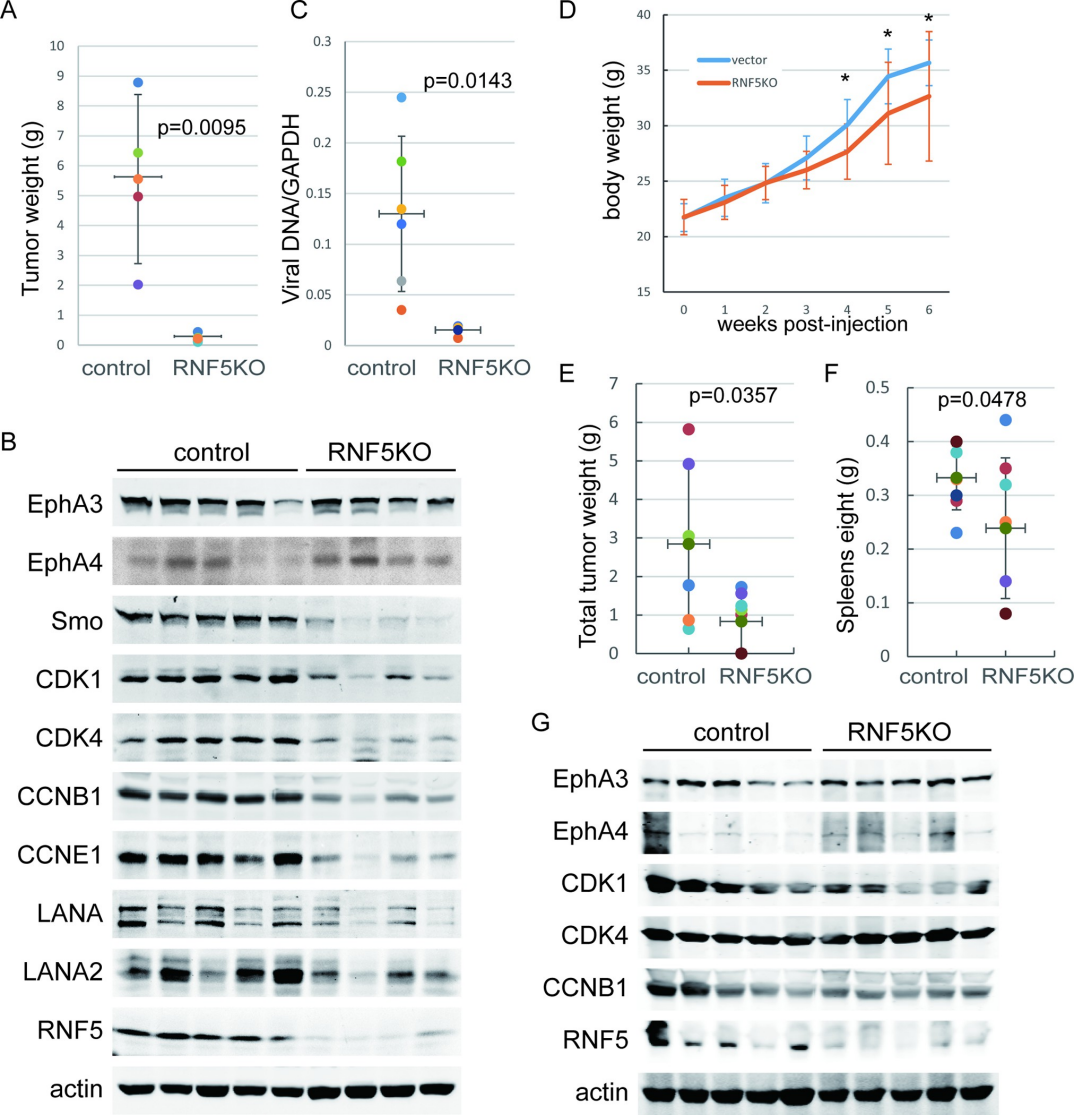
RNF5 loss decreases PEL xenograft tumor growth and cell cycle gene expression. A-C. RNF5 control or KO BCBL1 cells were subcutaneously injected into NOD/SCID mice for 6 weeks. A. After the mice were sacrificed, the tumors were collected, the tumor weights were measured, and the mean ± SD is shown. B. Whole extracts from random RNF5 WT or KO xenograft tumor samples were analyzed by western blots as indicated. C. Cellular and viral genomic DNA was isolated from tumors and detected by real-time PCR. The viral genomic DNA was normalized to GAPDH DNA, and the mean ± SD of relative levels is shown. D-G. RNF5 control or KO BCBL1 cells were intraperitoneally injected into NOD/SCID mice (8 mice per group) for 6 weeks. D. The body weights of mice were measured each week post-injection, and the curves of the mean ± SD are shown. *, p<0.05. E-F. After all mice were sacrificed, the effusions and invasive tumors in the peritoneal cavity and spleen were collected and measured. The total weight of the PEL pellets plus solid tumors of each mouse (E) and the weight of spleens (F) are shown. G. The whole extracts of cell pellets in effusions from 5 random RNF5 WT or KO BCBL1-injected mice were analyzed by western blots as indicated.

To further measure the effect of RNF5 inhibition on PEL tumorigenesis, we subcutaneously injected stable control BCBL1 cells or BCBL1 cells with RNF5 knockdown by shRNA or RNF5 KO using CRISPR-Cas9 into NOS/SCID mice and measured xenograft tumor growth in mice derived from these PEL cells. Both RNF5 silencing and RNF5 loss dramatically suppressed xenograft tumor growth (Figs 6A, 6B and 7A), suggesting that inhibition of RNF5 effectively suppressed tumor growth of primary effusion lymphoma.

To further investigate the mechanism of RNF5 inhibition in tumor suppression, we analyzed the global differential gene expression in these xenograft tumors by microarray analysis. Several pathways that are required for tumorigenesis, such as the hedgehog, MAPK and Ras pathways, were downregulated in shRNF5-transduced tumors (Fig 6C and S1 Table). Similarly, several inhibitory pathways of tumorigenesis, such as ECM-receptor interaction and focal adhesion, were upregulated in RNF5-knockdown tumors. By deep mining the differential transcriptional profiles and gene set enrichment analysis (GSEA) between scramble shRNA- and shRNF5-transduced PEL xenograft tumors, we found that the gene sets of the cell cycle (NES: -2.89, p-value: < 0.01) and hedgehog signaling (NES: 1.82, p-value: 0.0176) pathways were enriched by RNF5 silencing (Figs 6D and S5). Furthermore, we observed that the expression levels of Smo and multiple regulators of the cell cycle, such as CDK1, CyclinB1 and CyclinE1, were decreased in tumors with either RNF5 silencing or RNF5 loss compared to scramble or control tumors, respectively (Figs 6E and 7B).

In addition, the expression of EphA4 was increased in tumors with RNF5 silencing or loss compared with scramble or control tumors, respectively, while the expression of EphA3 was slightly increased (Figs 6E and 7B). These results suggest that RNF5 inhibition suppresses xenograft tumor growth by increasing EphA4 levels. Given that Ephrin receptors regulate the signaling pathways described above [9, 43], we conclude that increases in EphA4 may play key roles in the tumor suppression of RNF5 inhibition in primary effusion lymphoma.

As the expression levels of viral genes and the production of infectious viral particles are essential for viral persistence in KSHV-related neoplasms [44], we detected genomic viral DNA in tumors to address the influence of RNF5 KO on viral factors in vivo. We found that RNF5 KO decreased genomic viral DNA loads in BCBL1-derived subcutaneous tumors (Fig 7C), indicating that RNF5 KO decreased spontaneous lytic replication and genomic viral DNA loads during BCBL1-derived tumor growth. We further detected viral gene expression levels in RNF5-silenced or RNF5-loss xenograft tumors. The expression levels of the latent genes LANA and LANA2 were reduced in RNF5-silenced or RNF5 KO tumors compared with control tumors (Figs 6E and 7B), with undetectable levels of lytic gene expression in both types of tumors. These results indicated that RNF5 KO decreased genomic viral DNA loads during tumor formation, resulting in decreased LANA-positive cells and low LANA expression levels in RNF5 depleted or lost tumors. Since shRNF5 cannot completely deplete RNF5 expression while RNF5 KO does, the inhibition of host and viral gene expression by shRNF5 varied following RNF5 knockdown efficiency, while RNF5 KO generated a more profound inhibitory phenotype in tumors. These results suggest that RNF5 inhibition suppresses PEL with decreased KSHV latent gene expression levels and persistent infection.

To further investigate the inhibition of PEL growth in vivo by RNF5 loss, control or RNF5 KO BCBL1 cells were intraperitoneally injected into NOS/SCID mice, and then the cells grew as lymphoma in the abdominal cavity. The weight increase of mice injected with RNF5 KO cells was much slower than that of mice injected with control cells, which had obviously swollen abdomens (Fig 7D). After all lymphoma and invasive tumors in the abdominal cavity were collected and weighed, the total tumor weight was reduced in mice injected with RNF5 KO BCBL1 cells compared with mice injected with control cells (Fig 7E). The spleens of mice injected with control cells were swollen, while spleen enlargement was reduced in mice injected with RNF5 KO cells (Fig 7F). Furthermore, the expression level of EphA4 was mainly increased, while the expression levels of CDK1 and CCNB1 were decreased in the cell pellets of effusions from mice injected with RNF5 KO cells, and minor changes were observed in EphA3 and CDK4 levels (Fig 7G). These results suggest that RNF5 loss suppresses xenograft PEL growth in NOS/SCID mice, with increased EphA4 levels and decreased cell cycle gene expression.

**Fig 8:**
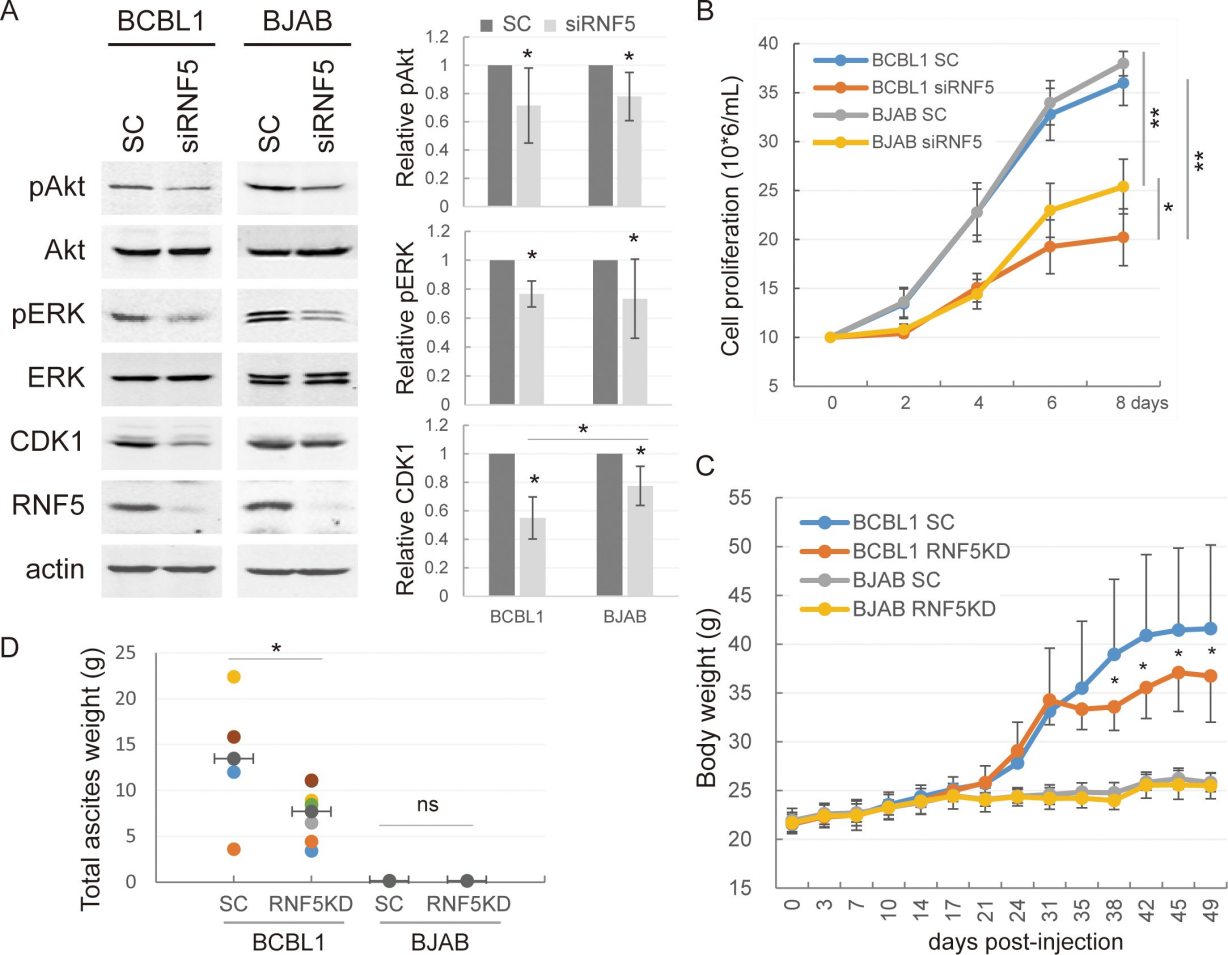
RNF5 knockdown preferentially suppresses the growth of PEL cells in vitro and in vivo. BJAB and BCBL1 cells were infected with scramble or siRNF5-expressing lentivruses and then the stable cells were selected with 200 μg/mL hygromycin for 2 weeks. A. Control or RNF5 KD BCBL1 and BJAB cells were harvested and cell extracts were analyzed by western blots as indicated. The relative levels of phosphorylation and expression were quantitated based on the intensity of grayscale of western blotting bands of two independent cell pools in duplicate. *, p<0.05. B. Control or RNF5 KD BCBL1 and BJAB cells were cultured in low serum medium containing 5% FBS, and cell numbers were recorded every days, and the growth curves are calculated from two stable cell pools in two independent experiments and shown. *, p<0.05; **, p<0.01. C-D. Control or RNF5 KD BCBL1 or BJAB cells were intraperitoneally injected into NOD/SCID mice (8 mice per group) for 7 weeks. C. The body weights of mice were measured twice each week post-injection, and the curves of mean ± SD are shown. *, p<0.05. D. After all mice were sacrificed, total lymphoma and ascites in BCBL1-injected mice and tumor-like tissues in the peritoneal cavity in BJAB-injected mice were collected. The total weights were recorded and shown. *, p<0.05; ns, no statistical difference.

To investigate whether RNF5 inhibition similarly affects the growth of KSHV-infected and non-KSHV-infected lymphoma cells, stable control vs. RNF5 knockdown (KD) BJAB and BCBL1 cells were established and their growth in culture and in NOD/SCID mice were measured. RNF5 depletion similarly suppressed Akt and ERK phosphorylation in both cells (Fig 8A), suggesting that RNF5 generally regulates Akt and ERK activation in KSHV-independent manner. The expression of CDK1 in both cells were also inhibited by RNF5 depletion, while the inhibition in BCBL1 cells was more significant than that in BJAB cells. Although control BCBL1 and BJAB cells had the similar curve of cell growth, RNF5 depletion decreased both cell growth in low serum condition, and the growth of RNF5 KD BCBL1 cells was much slower than RNF5 KD BJAB cells (Fig 8B), indicating that the expression of RNF5 plays more important roles in cell proliferation of KSHV-positive PEL cells than non-KSHV-infected lymphoma cells. When control and RNF5 KD BJAB or BCBL1 cells were intraperitoneally injected into NOS/SCID mice, all mice injected with control or RNF5 KD BCBL1 cells had swollen abdomens five weeks after injection, the weights of mice injected with RNF5 KD BCBL1 cells were decreased compared with the mice injected with control BCBL1 cells, while no significant difference of weights was observed in the mice injected with control vs. RNF5 KD BJAB cells that did not cause the swollen abdomens (Fig 8C). The total weights of BCBL1-derived lymphoma and ascites in the abdominal cavity were greatly reduced by RNF5 depletion when all mice were sacrificed seven weeks after injection, while no ascites or invasive tumors grew in mice injected with control or RNF5 KD BJAB cells, only small tumor-like tissues were observed in the abdominal cavity and RNF5 knockdown minimally decreased their weights (Fig 8D). These results suggest that KSHV-positive PEL cells exhibit more aggressive tumorigenesis than non-KSHV-infected lymphoma cells in NOD/SCID mice and that RNF5 silence generates more significant suppression in xenograft tumor growth of KSHV-positive PEL cells than non-KSHV-infected lymphoma cells.

## Discussion

**Fig 9:**
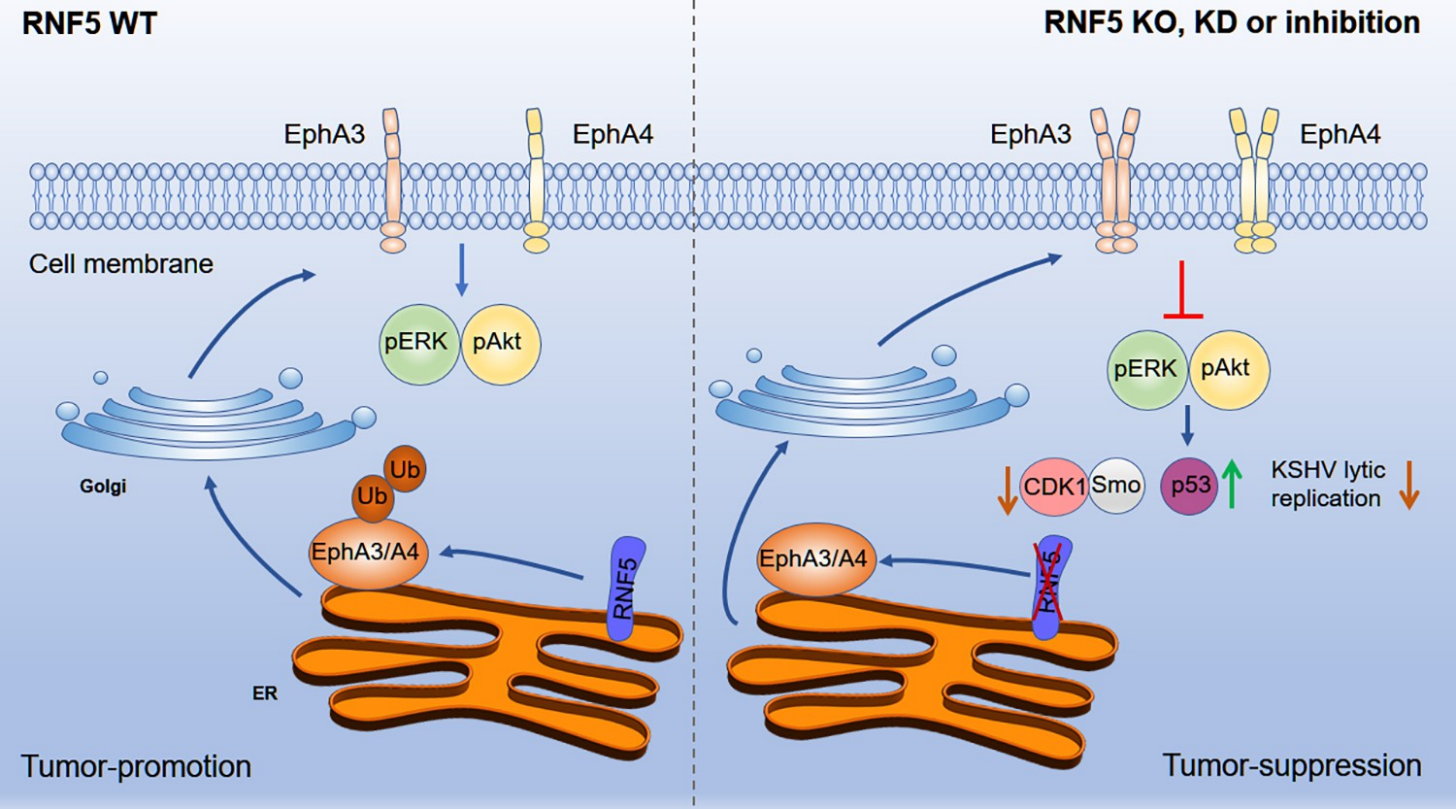
The diagram of RNF5 inhibition on KSHV lytic replication and PEL tumorigenesis. In wild-type PEL cells (left), RNF5 interacts with EphA3 and EphA4 and induces their ubiquitination and degradation, thereby limiting their levels in the cell membrane, in turn controlling ERK and Akt activation and facilitating KSHV lytic replication and PEL growth. In PEL cells with RNF5 silencing, loss, or inhibition (right), RNF5-mediated EphA3 and EphA4 ubiquitination and degradation are inhibited, and the levels of EphA3 and EphA4 are increased, probably facilitating dimerization and oligomerization, which decrease ERK and Akt activation and the expression of cell cycle CDK1 and hedgehog gene Smo, resulting in the inhibition of KSHV lytic replication and suppression of PEL growth.

Our current studies have shown that RNF5 interacts with two Ephrin receptors, EphA3 and EphA4, inducing their ubiquitination and degradation, and then inhibition of RNF5 by genetic approaches or inhibitors decreases ERK and Akt activation in PEL cells mainly through increased EphA3 and EphA4 levels and then decreases hedgehog signaling, cell cycle gene expression and KSHV lytic replication in PEL cells (Fig 9). Consequently, RNF5 silencing or RNF5 loss suppresses PEL-derived xenograft tumor growth, with increased EphA4 levels and decreased oncogenic KSHV viral gene expression and genomic viral DNA loads, providing a novel therapeutic candidate for KSHV lytic infection and KSHV-positive PEL treatment.

Genetic inhibition or chemical inhibitors of the PI3K-Akt and ERK-MAPK signaling pathways have been shown to significantly block KSHV lytic replication and reactivation from latency [31–36] and effectively suppress PEL tumorigenesis and KSHV pathogenesis [6, 45–47]; however, PEL cells might rapidly become insensitive or independent of these pathways because of complex feedback and/or crosstalk of cellular signaling [48–50], and the existence of viral products can cause drug resistance to these inhibitors, such as ORF45, which can contribute to U0126- or Rapamycin-insensitive ERK-RSK activation and eIF4B phosphorylation [33, 51]. Knowledge of complex upstream signaling and targets as well as the development of drug-insensitivity in PEL remains limited, and novel mediators and signal cascades need to be further investigated in KSHV lytic replication and PEL tumorigenesis. Our study showed that RNF5 inhibition suppresses PEL growth with two types of increased Ephrin type-A receptors, EphA3 and EphA4, and decreased activation of the ERK and Akt pathways, downregulating the expression of multiple cellular pathways and KSHV lytic viral genes, providing a novel choice for PEL therapy and drug development.

Although RNF5 regulates several key substrates involved in innate immunity, inflammation, and autophagy, we did not observe differential gene expression involved in these processes in RNF5 wild-type vs. RNF5-depleted BCBL1 cells and xenograft tumors (S1 Table), indicating that inhibition of RNF5 in PEL tumorigenesis was not due to these targets and pathways. Three types of Ephrin type-A receptors act as KSHV receptors [12, 13, 15]; however, EphA2 expression levels are undetectable in BCBL1 cells, whereas EphA3 and EphA4 are two highly expressed Ephrin type-A receptors. Our study revealed that RNF5 interacts with and induces EphA3 and EphA4 ubiquitination and degradation (Fig 3), subsequently regulating ERK and Akt activation and lytic gene expression through two novel substrates (Figs 4 and 5). Notably, depletion of EphA3 or EphA4 recovered ERK and Akt activation and lytic gene expression in RNF5 KO BCBL1 cells or RNF5-depleted iSLK.219 cells as well as the cell cycle-dependent kinase CDK1 and hedgehog gene Smo expression, indicating that these two novel substrates are involved in PEL suppression by RNF5 inhibition, and their interplay and function should be identified in further investigations to develop their potential application in PEL treatment.

Ephrin receptors can generate bidirectional roles in many important physiological processes in a ligand-independent or ligand-dependent manner; usually ligand-induced dimerization and oligomerization trigger suppressive signaling, while ligand-independent monomers exhibit promoting activity [43, 52]. In addition, the modification of ephrin receptors can regulate their functions, including phosphorylation and ubiquitination. In the present study, our results revealed that RNF5 loss, silencing, or inhibition increased EphA3 and EphA4 levels by blocking their ubiquitination and degradation, resulting in ligand-independent inhibition of ERK and Akt activation and KSHV lytic replication because neither EphrinA1 nor EphrinA4 treatment affects ERK and Akt activation and KSHV lytic replication in RNF5 WT vs. KO BCBL1 cells (S4 Fig). Probably the increased EphA3 and EphA4 levels in RNF5 KO cells may directly facilitate dimerization and oligomerization, or RNF5-mediated ubiquitination may block their dimerization and oligomerization. On the other hand, ligands trigger transient ERK and Akt activation and subsequent internalization of Ephrin receptors, whereas KSHV lytic replication requires prolonged ERK and Akt activation; thus, neither EphrinA1 nor EphrinA4 treatment is sufficient to trigger sustained ERK and Akt activation and regulate KSHV lytic replication. However, this phenomenon in cultured cells did not represent the function in vivo, in which frequent cell-cell contact and adhesion between PEL cells and lymphocytes or endothelial cells might cause different Ephrin receptor-ligand interactions and signal transduction.

The hedgehog signaling pathway is activated in B-cell lymphoma and plays an essential role in tumor growth and progression [53, 54]. Inhibition of Smo, a hedgehog receptor transducer, could be a therapeutic strategy for the treatment of diverse types of lymphoma [55, 56]. Increased EphA3 and EphA4 levels were observed under RNF5 inhibition and subsequently decreased ERK activation. Hedgehog signaling was downregulated by RNF5 inhibition and increased EphA3/EphA4 level, possibly through suppression of ERK activation, because ERK inhibition decreased Smo expression level in RNF5 WT cells, similar to RNF5 KO cells (S1 Fig). Studies have shown that EphA2 loss activates hedgehog signaling, which promotes cell proliferation [57], and in the present study, depletion of EphA3 or EphA4 recovered the decreased Smo and CDK1 expression in RNF5-loss or RNF5-silencing cells (Figs 4 and 5). These results suggest that EphA3 and EphA4 decrease hedgehog gene expression and reduce cell growth under RNF5 inhibition, probably through decreased ERK and Akt activation.

As PEL is etiologically associated with infection with oncogenic γ-herpesviruses, decreased ERK and Akt activation reduces viral gene expression and viral loads in PEL cells [37, 38, 58]; thus, selective RNF5 inhibition suppresses KSHV lytic gene expression and lytic replication, consequently attenuating or eliminating genomic viral DNA loads and persistent KSHV infection (Figs 6 and 7). In addition, RNF5 inhibition increased the expression of the tumor suppressor gene p53 (Figs 1 and 2). RNF5 inhibition suppresses PEL through multiple pathways, including both cellular and viral signaling, which usually act as potential targets for PEL treatment and drug development.

Studies have shown that spontaneous KSHV lytic replication is important for PEL tumorigenesis and has been demonstrated as a promising target for PEL treatment [7, 44]. Our results show that KSHV-positive PEL cells generate more aggressive tumorigenesis in NOD/SCID mice than non-KSHV-infected lymphoma cells, and RNF5 silence significantly suppresses xenograft tumor growth of KSHV-positive PEL cells in NOD/SCID mice, while slightly affects in vivo growth of non-KSHV-infected lymphoma cells (Fig 8C and 8D), because RNF5 inhibition not only suppresses tumor cell growth and tumorigenesis through multiple pathways, but also effectively disrupts KSHV lytic replication and consequent KSHV-induced the expression of cytokines and inflammatory factors. Thus we conclude that RNF5 inhibition might preferentially generate greater suppression in tumorigenesis of KSHV-positive PEL than KSHV-negative lymphoma.

Although RNF5 is ubiquitously expressed with low tissue specificity, RNF5 loss or inhibition does not generate abnormalities or obvious phenotypes. RNF5 loss does not greatly affect cell survival and proliferation while enhances the basal autophagy in MEFs and mice macrophage [19], cell death was not induced by RNF5 loss or depletion under normal physiological condition even though it regulates the cell mobility and endoplasmic reticulum-associated degradation [59, 60]. Our study did not observe that RNF5 KO cells generated the obvious phenotype on cell growth and death under normal culture condition, and enhanced the serum-dependence under low-serum culture condition instead (Fig 1B). Previous studies have also shown that no obvious phenotypic or pathological alterations were observed in RNF5 KO mice [19, 22]. In contrast, RNF5 inhibition results in enhanced autophagy and antitumor immunity, restricting bacterial infection and tumor growth [19, 22]. These studies reveal that RNF5 is not an absolutely essential fundamental E3 ligase in cell and body physiology that knocking it down or out always has some phenotype on cell survival or viability, even though it is ubiquitously expressed and negatively regulates several important processes, including innate immune and inflammatory responses and autophagy during viral and bacterial infection. A recent study also showed that RNF5 inhibition decreased the development and progression of acute myeloid leukemia (AML) [29]. These studies suggest that targeting RNF5 may generate a potent therapeutic benefit for lymphoma and leukemia treatment without obvious toxicity or damage. Thus, our study provides a promising candidate and approach for developing the treatment of KSHV-positive diseases and primary effusion lymphoma by selective RNF5 inhibition.

## Materials and methods

### Ethics statement

All animal experiments were conducted according to relevant guidelines and approved by the Animal Welfare and Ethics Committee of the Experimental Animal Center of Sun Yat-sen University.

### Cell lines

Primary RNF5 WT and KO MEFs were previously described [19] and cultured in DMEM supplemented with 10% fetal bovine serum (FBS) and 1% antibiotics (penicillin and streptomycin). The lymphoma lines BJAB, BC3, BCBL1, and JSC1 were maintained in our laboratory and cultured in suspension in RPMI 1640 medium containing 10% FBS and 1% antibiotics. HEK293T cells were cultured in DMEM supplemented with 10% fetal bovine serum (FBS) and 1% antibiotics. KSHV-BAC-harboring iSLK.219 cells were cultured in DMEM supplemented with 10% fetal bovine serum (FBS) and 1% antibiotics.

### Antibodies, chemicals, plasmids, and shRNA

The anti-RNF5 antibody and RNF5- and shRNF5-expressing plasmids have been previously described [19]. The HA-tagged RNF5 RM mutated plasmid was constructed as previously described [60]. The RNF5 inhibitor INH2 was a gift from Dr. Nicoletta Pedemonte at Istituto Giannina Gaslini, Italy [30]. Antibodies against p44/42 MAPK, p-p44/42 MAPK (Thr202/Tyr204), p-Akt (Ser473), Akt, and p53 (1C12) were purchased from Cell Signaling Technology (Danvers, MA, USA). Anti-EphA3 antibody was purchased from Genetex Inc. (Irvine, CA, USA). Antibodies against EphA4, CCNB1, CCNE1, CDK1, CDK4 and Smo were purchased from ABclonal Biotechnology Co. Ltd., China.

The shRNAs targeting EphA3 and EphA4 were subcloned into the pLKO.1 vector with puromycin resistance, and siRNF5 into the pSIREN-RetroQ vector (Clontech) with hygromycin resistance. The sequences are as follows:

shEphA3-1, CCTTCCAATGAAGTCAATCTA; shEphA3-2, GCCGCAAGTTTGAGTTTGAAA;

shEphA4-1, GACTTGCAAGGAGACGTTTAA; shEphA4-2, TCAGTCCGTGTGTTCTATAAA;;

siRNF5-1, GCGCGACCTTCGAATGTAA; siRNF5-UTR: CGGCAAGAGTGTCCAGTAT.

RNF5 gRNA was subcloned into the lentiviral vector pL-CRISPR.SFFV. GFP [61] and the sequences were as follows:

gRNF5-1, CAGTTGGCCATGTCTTCATC; gRNF5-2, AGACCAGCTCCGGAGAGCAG.

### Generation of BCBL1 RNF5 KO cell lines

BCBL1 cells were infected with pL-CRISPR. SFFV.GFP-based RNF5 gRNA-expressing lentiviruses (MOI = 0.1~0.5) for 48 h, and GFP-positive cells were sorted using BD Cell Sorter and seeded into 96-well cell culture plates (1–2 cells/well). After the cells were cultured for 3 weeks for expansion, the cells were normally passaged, and cell extracts were detected by western blotting to select the complete RNF5 knockout cells. At least three strains of RNF5 KO BCBL1 cells were cultured, frozen, and stored in liquid nitrogen.

### Cell viability and proliferation

PEL cell viability was detected using the CellTiter One Solution Cell Proliferation Assay (Promega, Madison, WI, USA) as described previously [39]. For PEL cell proliferation, the cells were diluted and stained with trypan blue, and living cells were analyzed using an automated cell counter. The cell cycle was analyzed by flow cytometry after cells were fixed with pre-cold 70% ethanol at 4°C overnight, permeabilized, digested with 0.1% Triton X-100 plus 100 ng/ml RNase A in PBS, and stained with 50 mg/ml propidium iodide (PI).

### Western blots and immunoprecipitation

Western blotting and immunoprecipitation assays were described previously [19]. Briefly, the cells were collected, washed once with cold PBS, and then lysed in lysis buffer (50mMTris-HCl, pH 7.4, 150 mM NaCl, 1% NP-40, 10% glycerol, 40 mM β-glycerophosphate, 30 mM sodium fluoride, 5 mM EDTA, 1 mM sodium orthovanadate) with complete protease inhibitor cocktail (Roche). For immunoprecipitation, the cell extracts were pre-cleared and then incubated with 2 μg primary antibodies overnight and then with protein G-agarose for 2 h at 4°C, the immunoprecipitated complexes were washed five times, and subjected to SDS-PAGE electrophoresis and immunoblotting analysis. For western blotting, the whole cell extract was denatured, separated by SDS-PAGE, and transferred to NC or PVDF membranes. The membranes were blocked and incubated with primary antibodies and species-matched secondary antibodies. The images were detected using the LI-COR Odyssey system or exposed to X-ray films using chemiluminescence.

### In vitro and in vivo ubiquitination assays

In vitro and in vivo ubiquitination were performed as previously described [19]. For in vitro ubiquitination, the bead-bound EphA3 or EphA4 proteins were affinity-purified with antibody and incubated with 200 ng/mL ubiquitin, 2 ng/mL E1 ligase, 20 ng/mL E2 ligase in a total volume of 25 μL for 60 min at 37°C, after which the bead-bound proteins were washed with washing buffer (50 mM Tris-HCl, pH 8.0, 150 mM NaCl, 0.2% Triton X-100, 0.1% SDS) and subjected to western blotting analysis with anti-ubiquitin antibody. For in vivo ubiquitination, the cells were collected and lysed with lysis buffer containing final 1% SDS and denatured at 95°C for 10 min. The lysates were diluted to a final concentration of 0.1% SDS, pre-cleared, and incubated with 2 μg of primary anti-EphA3 or anti-EphA4 antibody and 30 μL of protein A/G beads at room temperature. The beads were washed three times and subjected to SDS-PAGE and western blotting analysis with an anti-ubiquitin antibody.

### Induction of KSHV lytic replication and detection of lytic replication

To induce KSHV lytic replication, BCBL1 cells were treated with 20 ng/ml 12-O-tetradecanoylphorbol-13-acetate (TPA) and iSLK.219 cells were treated with 1 μg/mL doxycycline (Dox) plus 0.3 mM sodium butyrate (NaB). To detect lytic replication, cells were collected at different times after induction, cell extracts were quantitated and subjected to western blotting analysis with antibodies against viral proteins, or total RNA was extracted, reverse-transcribed, and detected by real-time PCR to detect viral mRNA levels. To detect the virion production, BCBL1 cells were induced for 4 days or iSLK.219 cells were induced for 5 days, and then the supernatants were collected and centrifuged twice at 12000 g to remove the cell debris, the supernatant was digested with DNaseI at 37°C for 1 h, stopped by EDTA plus SDS, followed by proteinase K digestion at 55°C for 30 min. Finally, virion DNA was extracted using a standard phenol-chloroform procedure and determined by real-time PCR.

### Preparation and infection of lentiviruses

The shRNA lentiviruses were prepared in 293T cells following the standard procedure described previously [62]. Briefly, 10 cm dish of 293T cells were co-transfected with 5 μg pLKO.1 shRNA plasmid, 5 μg psPAX2, and 5 μg pMD.2G packaging plasmids. After the cell culture medium was refreshed 6 h after transfection, the supernatants were harvested after an additional 48 h of incubation and stored as lentiviral stocks. After the lentiviruses were titrated, the cells were infected at an MOI = 5 in the presence of 4 μg/ml polybrene. At 6 h post-infection, the viral stocks were removed, and the cell culture medium was refreshed.

### Real-time PCR

Total RNA was extracted, reverse-transcribed, and analyzed by real-time PCR using the SYBR Green Real-Time PCR Master Mix Kit (Roche). Virion DNA and viral genomic DNA were isolated and detected by real-time PCR as described previously [63, 64]. The primers used for real-time PCR analysis were described previously [65] or designed using PrimerBank [66].

### Xenograft tumor growth

Five million stable BCBL1 cells with scramble or shRNF5 expression and control cells or cells with RNF5 loss by CRISPR/Cas9 were mixed with an equal volume of Matrigel (200 μL) and subcutaneously injected into NOD/SCID mice. When the largest tumors were up to 2 cm in diameter after 6 weeks, all mice were sacrificed and the tumors were collected. After the tumor size or weight was determined, the tumors were cut and fixed with formaldehyde solution for IHC analysis or frozen in liquid nitrogen for immunoblot and microarray analyses.

For body cavity fluid analysis, 1×10^7^ cells (BCBL1 or BJAB cells suspended in 200 μL PBS) were intraperitoneally injected into NOD/SCID mice, and the body weights of mice were measured each week. All mice were sacrificed 6–7 weeks post injection. The effusions and invasive tumors in the peritoneal cavity were collected and examined, and cell pellets in the fluids were collected by centrifugation at 300 × g for 5 min and washed once with cold PBS.

### Microarray and data analysis

Tumor samples frozen in liquid nitrogen were homogenized, and mRNA was extracted and analyzed using an Agilent Human GE 4 × 44 K microarray chip (Kangchen Biotech, Shanghai, China). The intensity was quantitated and normalized, and the differentially expressed genes were clustered by fold change (> 2) and p-value (<0.01). The microarray data of all gene expression and the differentially expressed genes were listed in supplementary S1 Table.

Signaling pathway enrichment was performed using gene set enrichment analysis (GSEA) and Kyoto Encyclopedia of Genes and Genomes (KEGG) enrichment analysis [67]. Briefly, the parameters were: gene sets database was c2.all.v2022.1.Hs.symbols.gmt (curated), permutation type was gene set, and metric for ranking genes was Ratio of Classes.

## Supporting information

S1 Fig ERK inhibition decreases CDK1 and Smo expression. RNF5 wild-type or KO MEFs were left untreated or treated with 5 mM DTT or 20 μM CQ for 4 h or 10 μM U0126 for 24 h. Then, the cells were collected and cell extracts were analyzed by western blotting as indicated.
(TIF)

S2 Fig The expression profile of Ephrin receptors and ligands in BCBL1 cells. Total RNA was extracted from BCBL1 cells, reverse-transcribed, and detected by real-time PCR; the levels of gene expression were normalized to GAPDH, and the relative levels are shown.
(TIF)

S3 Fig Quantification of protein levels in Figs 4A, 4B, 5E and 5F. The relative expression levels were quantitated based on the intensity of grayscale of western blotting bands and then normalized to the total proteins (pERK and pAkt) or actin level (cellular or viral proteins). A. Supplementary to Fig 4A and 4B. B. Supplementary to Fig 5E and 5F.
(TIF)

S4 Fig Neither EphrinA1 nor EphrinA4 recovers ERK and Akt activation and KSHV lytic replication in RNF5 KO BCBL1 cells. BCBL1 WT vs. KO BCBL1 cells were incubated with 1 mg/mL Fc, EphrinA1 or EphrinA4 protein daily and left untreated (A) or treated with TPA for 3 days. The cells were collected, and cell extracts were subjected to western blotting analysis to detect ERK and Akt phosphorylation (A) or viral lytic gene expression (B).
(TIF)

S5 Fig Supplementary to Fig 6A and 6B. A-B. The transcriptional profiles of the cell cycle and hedgehog genes in the scrambled vs. shRNF5-transduced xenograft tumors were enriched by GSEA. C. The expression levels were quantitated based on the intensity of grayscale of western blotting bands in Fig 6E, and the relative expression levels of these genes were normalized to the actin level. *, p<0.05.
(TIF)

S1 Table Differentially expressed cellular genes in scramble vs. shRNF5-transduced BCBL1-derived xenograft tumors. (XLSX)
