# Supplementary figures and images for: Suppression of KSHV lytic replication and primary effusion lymphoma by selective RNF5 inhibition
Source: PLoS Pathog. 2023 Jan 19;19(1):e1011103. doi: 10.1371/journal.ppat.1011103 (PMC9888681; doi:10.1371/journal.ppat.1011103)

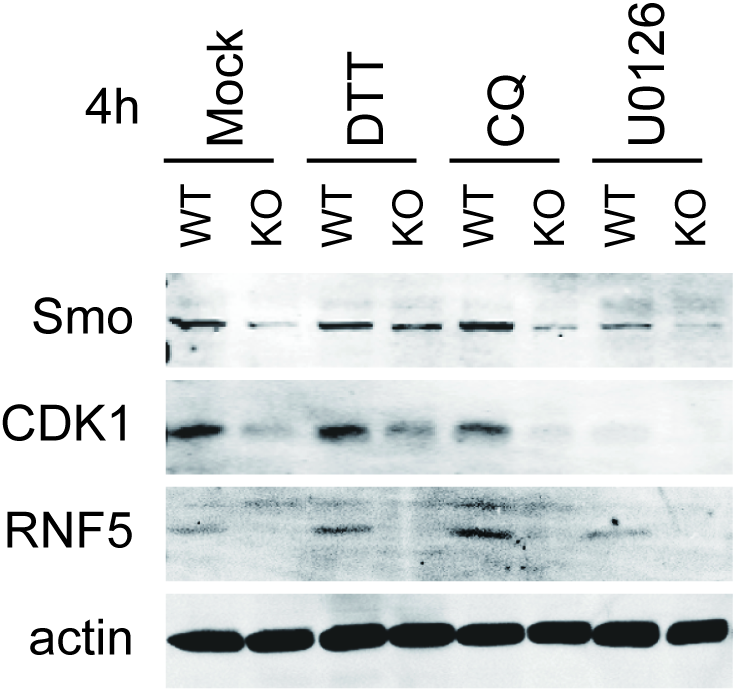

Supplement: S1 Fig — RNF5 wild-type or KO MEFs were left untreated or treated with 5 mM DTT or 20 μM CQ for 4 h or 10 μM U0126 for 24 h. Then, the cells were collected and cell extracts were analyzed by western blotting as indicated. (TIF) [file ppat.1011103.s001.tif]

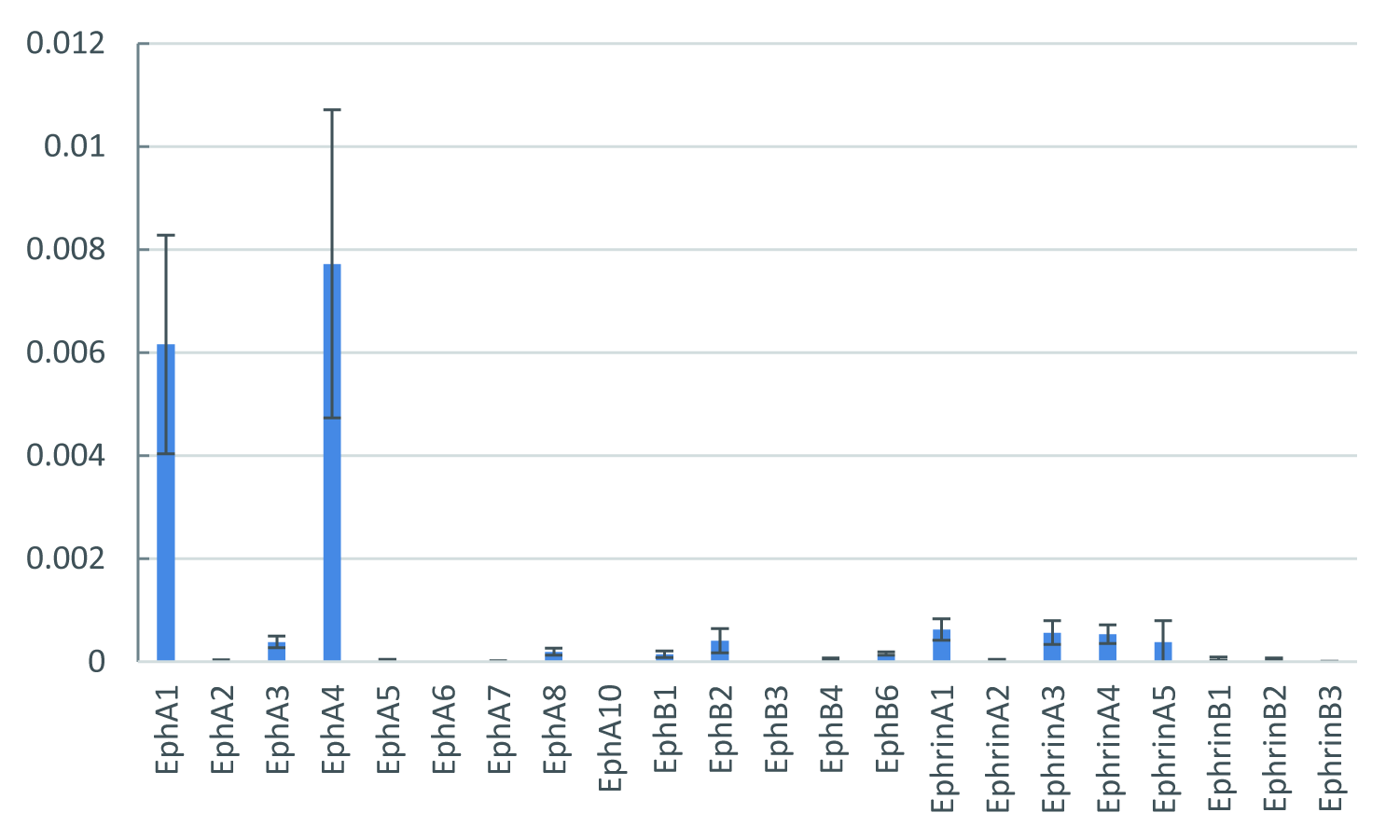

Supplement: S2 Fig — Total RNA was extracted from BCBL1 cells, reverse-transcribed, and detected by real-time PCR; the levels of gene expression were normalized to GAPDH, and the relative levels are shown. (TIF) [file ppat.1011103.s002.tif]

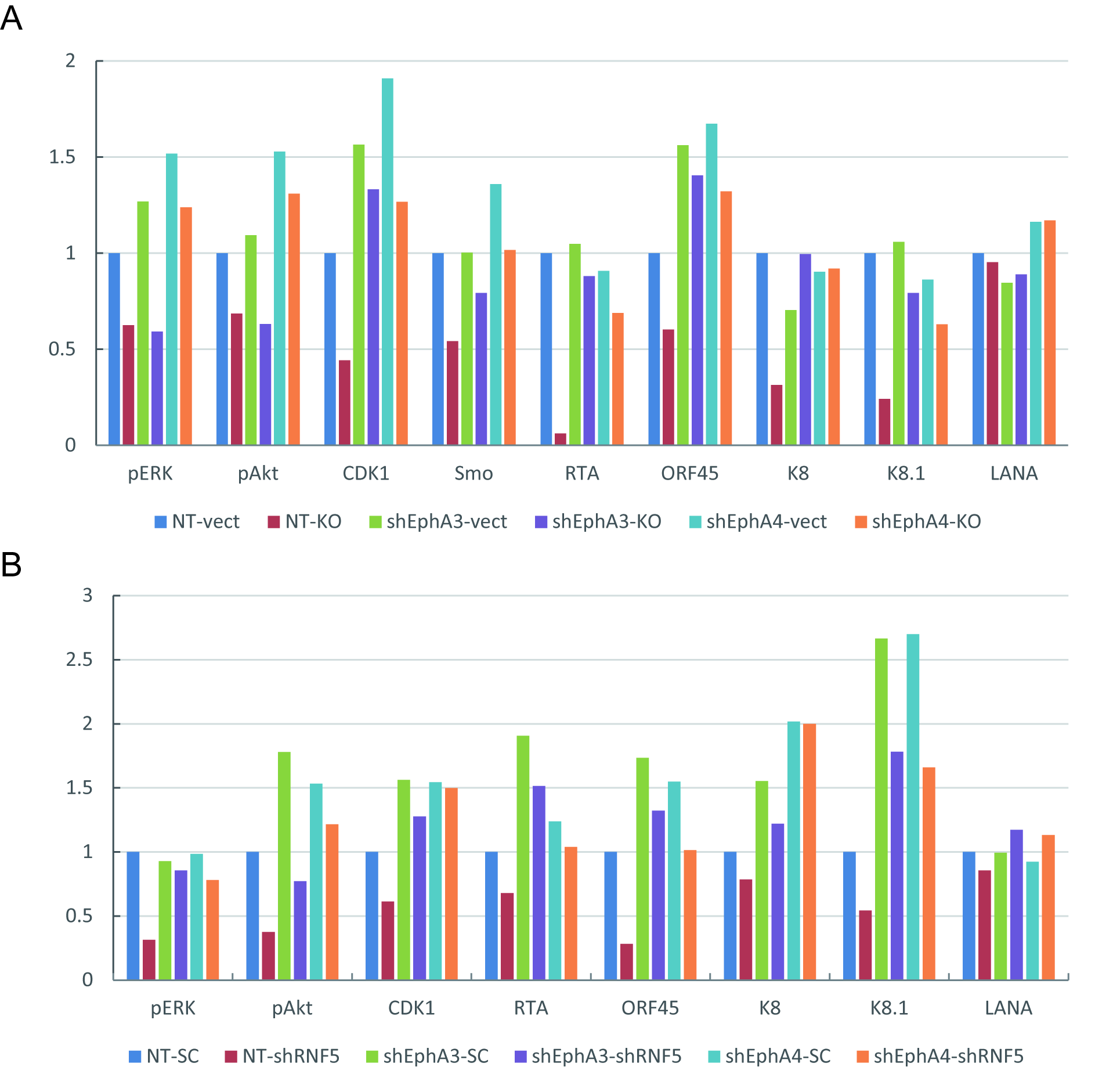

Supplement: S3 Fig — The relative expression levels were quantitated based on the intensity of grayscale of western blotting bands and then normalized to the total proteins (pERK and pAkt) or actin level (cellular or viral proteins). A. Supplementary to Fig 4A and 4B. B. Supplementary to Fig 5E and 5F. (TIF) [file ppat.1011103.s003.tif]

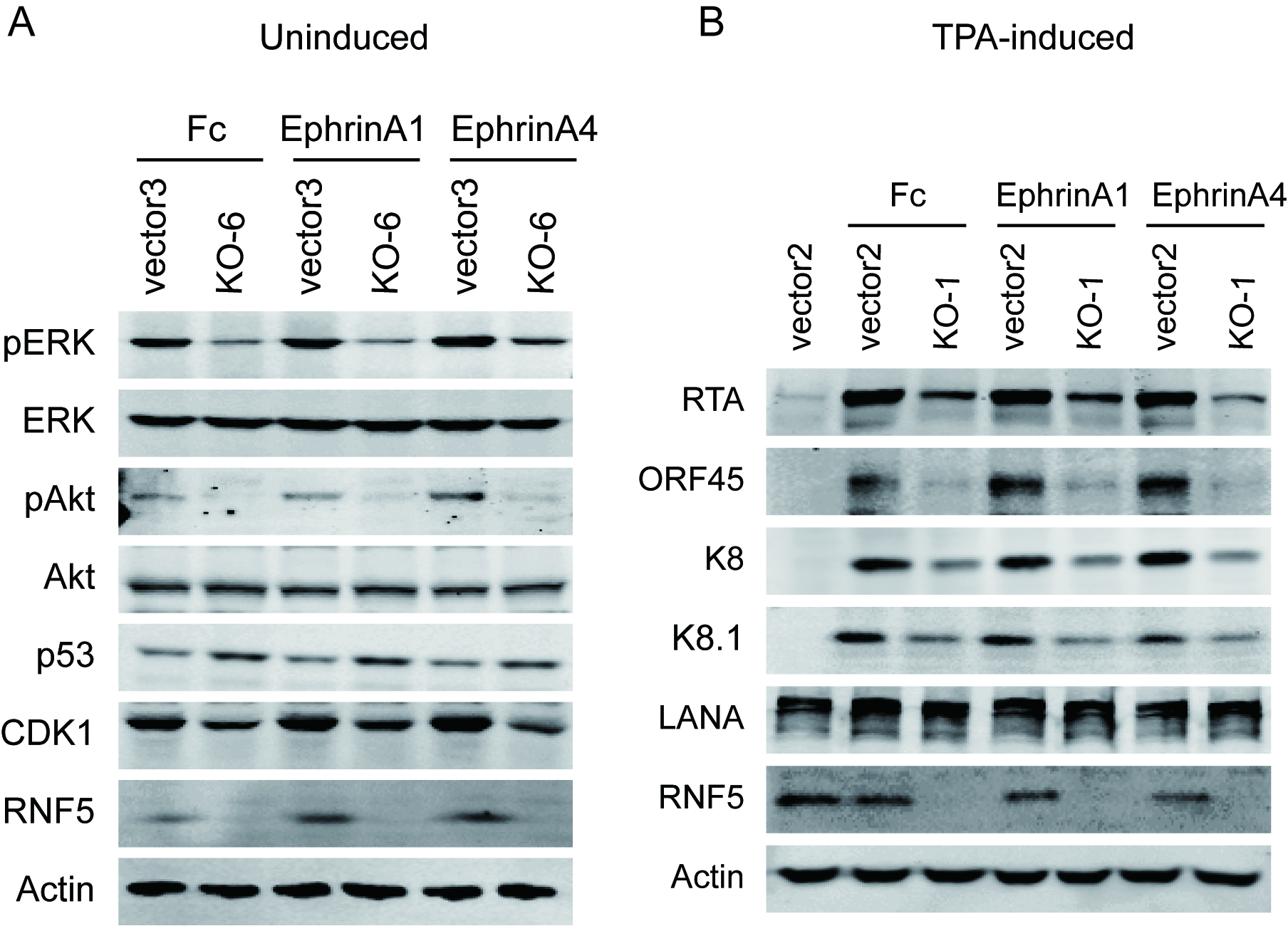

Supplement: S4 Fig — BCBL1 WT vs. KO BCBL1 cells were incubated with 1 mg/mL Fc, EphrinA1 or EphrinA4 protein daily and left untreated (A) or treated with TPA for 3 days. The cells were collected, and cell extracts were subjected to western blotting analysis to detect ERK and Akt phosphorylation (A) or viral lytic gene expression (B). (TIF) [file ppat.1011103.s004.tif]

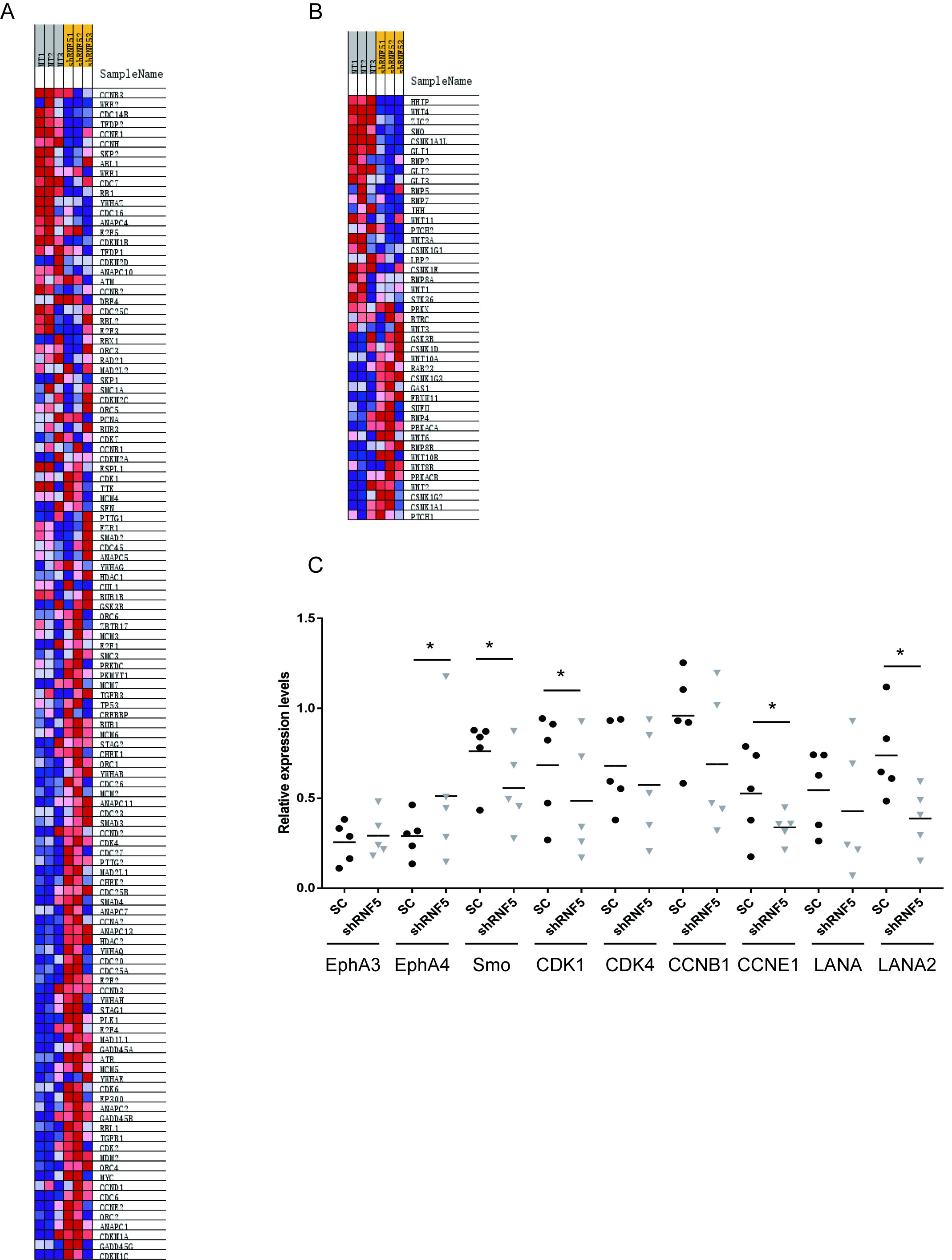

Supplement: S5 Fig — A-B. The transcriptional profiles of the cell cycle and hedgehog genes in the scrambled vs. shRNF5-transduced xenograft tumors were enriched by GSEA. C. The expression levels were quantitated based on the intensity of grayscale of western blotting bands in Fig 6E, and the relative expression levels of these genes were normalized to the actin level. *, p<0.05. (TIF) [file ppat.1011103.s005.tif]
